# Multi-omics driven computational framework for cancer molecular subtype classification

**DOI:** 10.1038/s41598-025-32051-5

**Published:** 2025-12-19

**Authors:** Ahtisham Fazeel Abbasi, Muhammad Sajjad, Muhammad Nabeel Asim, Sebastian Vollmer, Andreas Dengel

**Affiliations:** 1 Department of Computer Science, Rhineland Palatinate Technical University of Kaiserslautern-Landau (RPTU), Kaiserslautern, 67663 Rhineland-Palatinate Germany; 2 Smart Data and Knowledge Services (SDS), German Research Center for Artificial Intelligence (DFKI), Kaiserslautern, 67663 Rhineland-Palatinate Germany; 3 Data Science and its Applications (DSA), German Research Center for Artificial Intelligence (DFKI), Kaiserslautern, 67663 Rhineland-Palatinate Germany; 4 Intelligentx GmbH, (intelligentx.com), Kaiserslautern, 67663 Rhineland-Palatinate Germany

**Keywords:** Cancer, Computational biology and bioinformatics, Oncology

## Abstract

Cancer molecular subtype classification is an essential component of precision oncology which provides insights into cancer prognosis and guides targeted therapy. Despite the growing applications of AI for cancer molecular subtype classification, challenges persist due to non-standardized dataset configurations, diverse omics modalities, and inconsistent evaluation measures. These issues limit the comparability, reproducibility, and generalizability of AI classifiers across different cancers and hinder the development of robust and accurate AI-driven tools. This study performs comparative analyses of 35 unique AI classifiers across 153 datasets, covering 8 omics modalities and 20 different cancers. Particularly, it investigates 6 different research questions, and based on comprehensive performance analyses of the 35 AI classifiers it elucidates the research questions with the following answers: (i) out of 17 different configurations for 5 out of the 8 tested omics modalities, RPPA (RPPA), Gistic2-all-data-by-genes (CNV), HM27 (Meth), and HiSeqV2-exon (Exon) configurations consistently yield better performance; (ii) in terms of 8 omics modalities, RNASeq, miRNA, CNV, and Exon generally achieve higher macro-accuracy (MACC) compared to Meth., Array, SNP and RPPA; (iii) SNP and RPPA modalities are prone to biases due to technical noise; (iv) traditional machine learning (ML) models (SVM, XGB, HGB) perform best on small and low-dimensional datasets, while deep learning (DL) models (ResNet18, CNN, NN, MLP) excel on large and high-dimensional datasets; (v) SVM achieves the highest mean MACC across all classifiers, with NN, ResNet18, DEEPGENE, and MLP also demonstrate strong performance; and (vi) DL classifiers show superior MACC as compared to ML classifiers in 12 out of 20 cancers. The findings offer key insights to guide the development of standardized, robust, and efficient AI-driven pipelines for cancer molecular subtype classification. This study enhances reproducibility and facilitates better comparison across AI methods, ultimately advancing precision oncology.

## Introduction

Cancer is a group of diseases characterized by uncontrolled growth and spread of cells that can invade nearby tissues and other parts of the body (metastasis)^1^. According to the World Health Organization (WHO), more than 200 cancers^2^ have been identified with different characteristics, causes, and symptoms^3^. These cancers are categorized based on their origin, structure, and molecular characteristics into several major groups: Carcinomas, Sarcomas, Leukemias, Lymphomas, Neuroendocrine Tumors, and mixed Tumors that vary significantly in terms of origin and mechanism of action^4^.

Cancers claim almost 10 million lives each year, and around 19.3 million new cancer cases are diagnosed, making it the second most common cause of death worldwide^5^. These high mortality rates and diagnostic challenges stem from its complex nature and molecular diversity since each cancer presents unique characteristics that complicate diagnosis and treatment^6^. For instance, as multiple cancers are asymptomatic in their early stages, symptoms often mimic other common conditions, and each cancer can have various molecular subtypes requiring distinct treatment approaches. Moreover, the same cancer type can behave differently in individual patients^7^ which highlights the critical need to go beyond cancer detection and address the complexities of understanding molecular subtypes and behaviors.

**Fig. 1:**
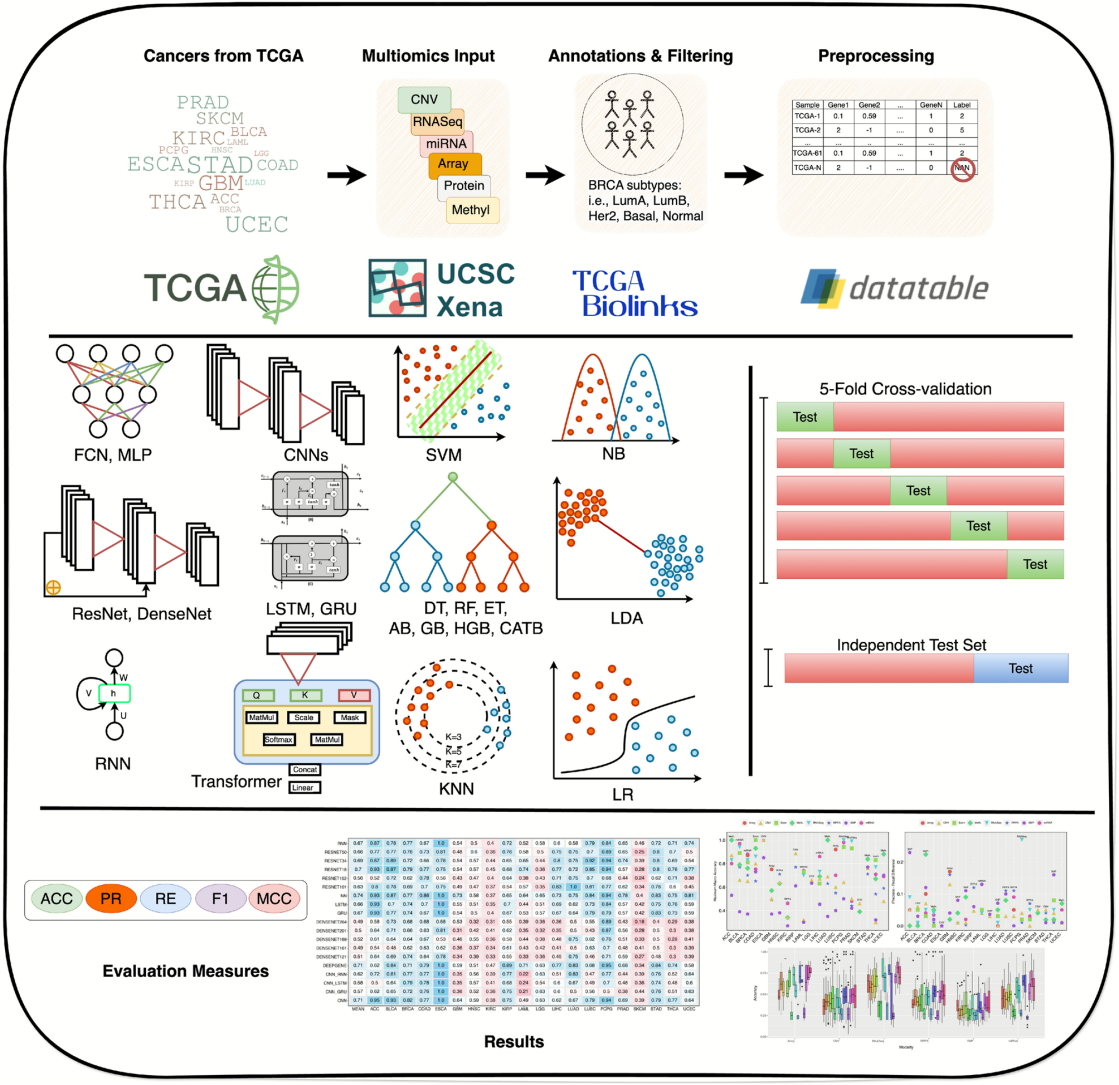
Graphical illustration of the cancer molecular subtype classification benchmarking framework, showcasing dataset preparation, AI classifiers, data splitting strategies, and evaluation methods. In the dataset preparation stage, omics data from TCGA are annotated, filtered, and preprocessed using tools such as UCSC Xena and TCGAbiolinks, covering omics modalities (CNV, RNASeq, miRNA, SNP, RPPA, and methylation). Next, a diverse set of AI classifiers, including 16 ML (e.g., SVM, RF, DT, and AB) and 18 DL (e.g., CNNs, ResNets, DenseNets, LSTMs, and transformers), classifiers are employed. Data splitting is performed using 5-fold cross-validation and independent test sets to ensure robust evaluation. Key performance metrics, including MACC, precision (PR), recall (RC), F1-score, Matthews correlation coefficient (MCC), with macro-averaging are applied for comparative analysis across all datasets.

The complexity of understanding cancer molecular subtypes led to The Cancer Genome Atlas (TCGA)^8^, a landmark project that revolutionized the understanding of cancer’s molecular basis^9^. TCGA has comprehensively mapped the genomic changes in major cancer types, revealing distinct molecular subtypes within what were previously thought to be single diseases. For example: Breast cancer has been classified into at least four major molecular subtypes (Luminal A, Luminal B, HER2-enriched, and Basal-like) Glioblastoma has been categorized into Classical, Mesenchymal, Neural, and Proneural subtypes. These molecular subtypes explain why patients with seemingly similar cancers can have vastly different outcomes and treatment responses.

Traditionally, cancer molecular subtypes are identified through a combination of wet lab experiments and computational analyses. Wet lab experiments involve techniques like genomic and RNA sequencing, epigenomic profiling, and proteomics to generate raw biological data from patient samples^10^. These methods provide valuable insights into genetic mutations, gene expression patterns, regulatory modifications, and protein levels, which are essential for understanding the molecular characteristics of different cancer molecular subtypes. In addition, computational methods such as clustering algorithms, and multi-omics integration are used to identify cancer molecular subtypes and correlate them with clinical outcomes^11^. Although both approaches are valuable for exploring cancer molecular subtypes, wet lab experiments are costly, time-consuming, and prone to variability, while traditional computational tools face challenges with scalability, noise sensitivity, and limited multi-omics integration.

Following the limitations of traditional methods and success of Artificial Intelligence (AI) in various application areas, i.e., omics^12^, genomics^13–16^, and proteomics^16–18^, the development of robust AI-based approaches for cancer molecular subtype prediction is an active area of research. Over the last two years, more than 60 AI-based approaches have been developed^19–21^ to predict cancer molecular subtypes more precisely. Among 60 studies, 15 studies have primarily focused on breast cancer molecular subtypes i.e., Basal-like, Luminal A, Luminal B, and HER2-enriched^22–24^. This prominence is driven by breast cancer’s high prevalence and the public availability of diverse, and well-curated datasets. Lung cancer ranks second, with 10 studies on subtypes like adenocarcinoma, squamous cell carcinoma, and large cell carcinoma^25–27^. The remaining studies are distributed across various cancers, including glioblastoma, colorectal cancer, gastric cancer, and leukemia, often tailored around specific subtypes^28–31^.

In terms of datasets, TCGA has been the most frequently utilized resource in over 30 studies for multi-omics data^25,32–34^. Other notable sources include the Gene Expression Omnibus (GEO)^35^, which has been utilized in 12 studies^36–38^, and cBioPortal in 8 studies^39–41^. Classical ML classifiers, such as Support Vector Machines (SVM), Random Forest (RF), and Logistic Regression (LR), have been used in approximately 25% of studies^42–45^. However, DL classifiers have been used in over 50% of studies. Models such as Convolutional Neural Networks (CNNs) and Graph Neural Networks (GNNs) have demonstrated superior performance in handling high-dimensional and multi-modal datasets^22,46^. In order to deal with the challenges of high dimensionality and noise, over 20 studies have reported the use of methods such as Principal Component Analysis (PCA), LASSO regression, Variational Autoencoders (VAEs), and t-SNE^47–50^.

Despite significant advancements in AI-based tools for cancer molecular subtype classification, existing studies often focus on individual cancers and offer cancer-specific insights that fail to generalize across diverse cancers. In addition, inconsistent evaluation i.e., varying metrics and disparate preprocessing strategies compromise the comparability and reproducibility of results across different studies. Moreover, the heterogeneity in omics modalities and configurations coupled with varied dataset sizes, hinder the development of standardized and robust tools for cancer molecular subtype classification. Furthermore, to the best of our knowledge, there is no existing study that thoroughly evaluates the predictive performance of AI classifiers for cancer molecular subtype classification by considering the challenges of generalizability across diverse cancer types, standardization of evaluation metrics, and the use of omics data with heterogeneous configurations and varying dataset sizes.

In light of these challenges, there is a pressing need for a comprehensive benchmarking framework that covers multiple cancer types, omics modalities and configurations, and diverse AI classifiers. To address this gap, we present a large-scale benchmarking study that evaluates the predictive performance of 35 ML and DL classifiers, across 153 datasets spanning 8 distinct omics modalities (miRNA, RNASeq, Exon, CNV, SNP, RPPA, Methylation, and Array) in terms of 20 different cancers. The objective of this benchmark is to delve into diverse aspects of the cancer molecular subtype classification, extract and furnish useful insights from diverse experiments with the following different research questions (RQs) and objectives: RQ I) What data configurations are critical for accurate cancer molecular subtype classification across 8 distinct omics modalities? RQ II) How consistently do different omics modalities perform in subtype classification across diverse cancers? RQ III) Are there specific omics modalities that lead to biased predictions, and what factors contribute to this bias? RQ IV) What ML and DL classifiers demonstrate reliable performance across all omics modalities? V) Which specific ML and DL classifiers provide consistent predictive performance with respect to cancers? RQ VI) Among top-performing classifiers in specific cancers, how do ML and DL methods compare in terms of consistency and suitability for cancer molecular subtype classification? RQ VIII) Do the conclusions drawn in RQ I, II, and III remain consistent when evaluated on an independent external validation dataset? We believe answers to these questions will offer critical insights for the research community in identifying the optimal combination of omics modalities, data configurations, and AI classifiers for the development of standardized, robust, and efficient end-to-end predictive pipelines for cancer molecular subtype classification.

## Materials and methods

This section briefly demonstrates the details of benchmark datasets, ML and DL classifiers, and evaluation measures. A high level graphical illustration of all three core components is given in Fig.  1 and briefly described in the following subsection.

### Summary of cancer molecular subtype classification benchmark framework

In recent years, the domain of precision oncology has seen a surge of omics-related datasets for cancer molecular subtype classification. These datasets span multiple omics modalities and are carefully curated to address challenges such as noise, and heterogeneity in molecular profiles. A detailed overview of similar datasets used in this study, spanning 8 distinct modalities and multiple configurations, is provided in section “Datasets”.

For classification tasks, within the different fields including natural language processing (NLP), computer vision (CV), and genomics, proteomics sequence analysis, ML has witnessed the development of 15 different types of classifiers, namely Naive Bayes (NB) (competent, Bernoulli), Decision Tree (DT)^51^, RF^52,53^, Gradient Boosting (GB)^54^, Extreme Gradient Boosting (xGB), Histogram-Based Gradient Boosting (HGB)^55^, Adaptive Boosting (AB)^56^, Light Gradient Boosting Machine (LGBM)^57^, Categorical Boosting (CB)^58^, K-Nearest Neighbors (KNN)^59^, SVM^60^, Quadratic Discriminant classifier (QDA)^61^, and Logistic Regresison (LR)^62,63^. Similarly, the DL field has witnessed multifarious architectures built upon core models such as CNNs^64,65^, Recurrent Neural Networks (RNNs)^66^, and their combinations, i.e., hybrid models. In addition, transformer-based architectures have emerged as promising architectures based on attention mechanisms to capture long-range dependencies and context in data. The primary objective of this study is to explore the potential of the aforementioned AI classifiers for cancer molecular subtype classification. To achieve this, we conducted in-depth experiments using various ML and DL classifiers. A detailed description of the ML and DL classifiers utilized in this study is provided in the following section “Classifiers”.

In order to evaluate the performance of AI-classifiers in the multi-class classification paradigm, 5 evaluation measures are commonly utilized. These evaluation measures encompass MACC, precision (PR), recall (RC), F1-score (F1), and Matthews correlation coefficient (MCC)^67^. In our study, AI classifiers are evaluated using these 5 evaluation measures which are briefly discussed in section “Evaluation measures”.

### Datasets

In the pursuit of establishing a robust foundation for the benchmark study of cancer molecular subtype classification, the selection of appropriate datasets plays a pivotal role. The choice of datasets directly impacts the accuracy, robustness, and generalizability of AI classifiers. Poorly selected datasets can result in inappropriate comparisons, biased models, misleading insights, and unreliable decisions in clinical or research settings. Therefore, it is imperative to select datasets that are both well-annotated and representative of the complex nature of cancer molecular subtypes.

Among publicly available resources for cancer molecular subtypes, TCGA offers a comprehensive collection of multi-omics datasets that encompasses a broad spectrum of cancer types and subtypes. It is chosen for two key reasons: first, it provides extensive diversity and coverage of cancers and subtypes, which are essential for training and evaluating AI classifiers; second, the datasets are publicly available, well-annotated, and rigorously curated, making them an ideal resource for benchmarking ML and DL classifiers^68–70^.

Supplementary file 1 Table S1 shows 153 benchmark datasets spanning 8 omics modalities and 17 unique configurations, in terms of 20 cancers which are collected from TCGA using UCSC Xena browser. The omics modalities include copy number variation (CNV), gene expression (RNASeq), microRNA expression (miRNA), single-nucleotide polymorphisms (SNPs), DNA methylation (Meth.), Exon expression (Exon), protein expression (RPPA) and Array. Each modality captures distinct biological features, contributing to the comprehensive characterization of cancer molecular subtypes. A detailed description of these modalities, corresponding configurations, and their technical characteristics are summarized in Table 1. In order to ensure accurate mapping of samples to labels, each dataset is processed using TCGABiolinks to map samples to their corresponding cancer subtype^71^. Datasets with fewer than 70 samples are excluded to ensure sufficient sample size for meaningful analyses and reliable model evaluation.

The selected datasets encompass a diverse range of sample sizes and subtype distributions, which reflect the inherent variability in cancer molecular subtype classification. On average, each dataset includes approximately 328 samples, with BRCA being the most samples, i.e., 1,097, while ACC represents the lower end of the spectrum with only 91 samples. This disparity underscores the challenge of modeling rare cancers effectively. In addition, there is a subtype imbalance across all datasets. For instance, in BRCA, the Luminal A subtype has 566 samples, whereas the Normal subtype is significantly underrepresented with just 40 samples. Similarly, in PRAD, the ERG subtype encompasses 152 samples, while FOXA1 accounts for only 9. In addition to differences in sample sizes and subtype distributions, the datasets exhibit considerable variability in features across modalities, which impacts model design and computational requirements. Meth. datasets are the most feature-rich, containing an average of 321,770 features, followed by SNP/INDEL datasets with 40,543 features and RNASeq datasets with 19,552 features. CNV datasets have a moderate feature count of 24,776 on average, while RPPA datasets are notably compact, averaging just 159 features. Given the extensive feature space of methylation datasets, these datasets undergo preprocessing before their use in classification. The details of the preprocessing steps are provided in Supplementary File 1.

Table 2 summarizes the 4 omics modalities obtained from the Metabric breast cancer cohort^72^ that are used for external validation in this study. Each modality, i.e., RNASeq, WES_WGS, CNV, and Meth., is represented with its corresponding platform or configuration, and information about classes and features. Together, these datasets provide a comprehensive and heterogeneous external benchmark for evaluating the validity of RQ I-III across distinct technologies and feature spaces.

**Table 1:** Description of diverse configurations of 8 distinct omics modalities.

Data modality	Summary	Scales	Technical information
CNV	Copy Number Variation (CNV) datasets in TCGA (The Cancer Genome Atlas) contain information about alterations in the number of copies of genomic regions, which can influence gene expression and drive cancer progression. CNVs include amplifications (gain of copies) and deletions (loss of copies) in DNA segments^73^	Gistic2 all thresholded	It contains thresholded CNV data produced by the GISTIC2 algorithm^74^. This dataset simplifies the raw copy number values into discrete levels, such as – 2 for homozygous deletions, – 1 for single-copy deletions, 0 for normal diploid regions, 1 for low-level amplifications, and 2 for high-level amplifications^75^. It aims to focus on significant genomic regions that are recurrently amplified or deleted across multiple cancer samples
		Gistic2 all databy genes	It provides gene-specific CNV information derived from the GISTIC2 analysis. Instead of reporting alterations at the regional level, this dataset maps CNV values directly to individual genes. The data often includes raw or continuous copy number values, offering finer detail about how specific genes are affected by CNVs
RNAseq	This dataset contains raw or normalized gene expression values derived from RNA sequencing (RNAseq) data produced using the Illumina HiSeq platform. The values typically represent either normalized counts, such as log-transformed reads per kilobase of transcript per million (RPKM) or fragments per kilobase of transcript per million (FPKM), or other forms of normalization^76,77^	HiSeqV2	It contains raw or normalized gene expression values derived from RNA sequencing (RNAseq) data produced using the Illumina HiSeq platform. The values typically represent either normalized counts, such as log-transformed reads per kilobase of transcript per million (RPKM) or fragments per kilobase of transcript per million (FPKM), or other forms of normalization^78^
		HiSeqV2 percentile	It provides gene expression values transformed into percentiles. Each gene’s expression level in a sample is ranked relative to other genes in that sample, and the rank is converted into a percentile value (0–100). This normalization removes inter-sample variability due to sequencing depth or other technical factors, allowing for comparisons of gene expression patterns across samples^71^
		Gav2	It contains raw and normalized gene expression values derived from RNAseq data generated using the Illumina HiSeq platform. The GAV2 pipeline employed MapSplice for read alignment and RSEM for transcript quantification against the hg19 (GRCh37) reference genome. The resulting values represent either raw read counts or normalized measures such as fragments per kilobase of transcript per million mapped reads (FPKM) and upper-quartile normalized FPKM (FPKM-UQ), providing gene and isoform-level expression estimates
Exon	It contains expression measurements at the exon level, derived from RNA sequencing data produced using the Illumina HiSeq platform. Instead of summarizing reads across entire genes, the exon dataset quantifies read counts mapped to individual exons, enabling finer-resolution analysis of alternative splicing, isoform usage, and exon-specific expression changes	GAV2	The GAV2 pipeline utilizes MapSplice for accurate spliced alignment and RSEM for transcript quantification against the hg19 (GRCh37) reference genome. It outputs exon-level read counts and normalized expression metrics such as FPKM and upper-quartile normalized FPKM. This dataset is particularly valuable for characterizing differential exon usage, identifying alternative splicing events, and exploring exon-specific expression alterations in tumors
		HiSeqV2	The HiSeqV2 Exon pipeline aligns reads to the reference genome using MapSplice and quantifies exon expression with RSEM. The resulting data are expressed as fragments per kilobase of exon per million mapped reads (FPKM). These exon-level profiles enable detailed analyses of isoform usage, exon inclusion/exclusion, and transcript diversity within cancer samples
miRNA	It contains expression values specifically for the mature strands of miRNAs. miRNAs are transcribed as precursor molecules that are processed into mature miRNA strands, which are the functional forms involved in gene regulation^79^. This dataset focuses exclusively on these mature miRNA strands, which directly interact with mRNA targets		
SNP and INDEL	This dataset encompasses all somatic mutations identified in tumor samples, including single-nucleotide polymorphisms (SNPs) and small insertions or deletions (INDELs). These mutations represent changes in the DNA sequence that occur in tumor cells and are absent in matched normal cells	Mutation wustl gene	This is a gene-level summary of somatic mutation data (SNPs and INDELs), processed and annotated by the McDonnell Genome Institute at Washington University (WUSTL)^80^. Instead of reporting individual mutations, this dataset aggregates mutations for each gene, providing an overview of the mutation burden and functional impacts on specific genes
		MC3 gene-level	The MC3 gene-level somatic mutation data^81^ from TCGA provides a comprehensive overview of mutations (SNPs and INDELs) across various cancer types, aggregated at the gene level. It includes details on point mutations (SNPs), where a single nucleotide is replaced, and insertions or deletions (INDELs) that alter the DNA sequence. The dataset contains information on mutations in thousands of genes across hundreds of cancer samples. Specifically, it includes mutation data for over 20,000 genes
RPPA	This refers to the measurement of the abundance of proteins in cancer samples. The data is typically obtained through techniques such as mass spectrometry or antibody-based assays, providing insights into the levels of different proteins in tumor tissues compared to normal tissues	RPPA	The Reverse Phase Protein Array (RPPA) technique is used to measure protein levels in cancer samples. RPPA provides quantitative data on protein expression, focusing on specific proteins or signaling pathways that are often involved in cancer progression^82^. The RPPA data in TCGA allows for the analysis of protein expression across multiple cancer types, providing a high-throughput method for assessing large numbers of samples
		RPPA-RBN	The RPPA-RBN (Reverse Phase Protein Array – RNA-Based Normalization) involves normalizing the RPPA data using RNA expression data to account for variations in sample quality and other factors. This normalization helps to refine protein expression analysis, ensuring that the observed protein levels are not biased by underlying RNA expression differences^83^
Meth.	It primarily captures methylation at cytosine residues in CpG dinucleotides, which can influence gene expression and chromatin structure. TCGA methylation data is generated using platforms like the Illumina HumanMethylation450 BeadChip (450K array) and, in some cases, the HumanMethylation27 BeadChip. Methylation is quantified as beta values, ranging from 0 (unmethylated) to 1 (fully methylated), allowing for analyses of hypermethylation and hypomethylation patterns	Human Methylation-450	It contains DNA methylation levels measured at over 450,000 CpG sites using the Illumina HumanMethylation450 BeadChip platform. Methylation levels are typically reported as beta values, ranging from 0 (unmethylated) to 1 (fully methylated)^84^. The dataset provides insights into epigenetic regulation by covering CpG sites in promoters, gene bodies, intergenic regions, and CpG islands, as well as regulatory regions like shores and shelves
		Human Methylation-27	The HumanMethylation27 dataset refers to a DNA methylation array platform that measures the methylation status of approximately 27,000 CpG sites across the human genome. The array is designed to assess the methylation patterns at these CpG sites, which are crucial in gene regulation^85^
Array	The Gene Expression Array datasets listed are part of TCGA’s collection, used to measure gene expression across various cancer types using different array platforms	AgilentG4502A07(1-3)	This dataset represents gene expression data obtained from the Agilent G4502A platform. The Agilent arrays use microarray technology to measure gene expression by hybridizing labeled RNA to probes on the array. The G4502A is one of Agilent’s arrays designed for high-throughput gene expression analysis, offering broad coverage of human genes. The different versions (e.g., 07-1, 07-2 and 07-3) may reflect updates to the array design or slight variations in the data, ensuring that the data from multiple sources or experiments is comparable^86^
		HTHG-U133A	This dataset refers to gene expression data generated using the Affymetrix Human Genome U133A Array. The U133A array is widely used in gene expression profiling and measures the expression of around 22,000 genes. It is a standard platform for large-scale studies in cancer genomics and transcriptomics, providing reliable data for identifying gene expression patterns across various samples

This diverse and curated dataset collection provides a solid benchmark for evaluating AI classifiers in cancer molecular subtype classification. It enables a comprehensive assessment of how well AI classifiers address the challenges posed by data heterogeneity, subtype imbalance, and modality-specific complexity.

**Table 2:** Summary of the metabric breast cancer cohort used for external validation.

Cancer	Modality	Configuration	Classes	Total no. of samples	Samples per class	No. of features
BRCA	RNASeq	Illumina HT-12 v3 Expression BeadChip	5	1756	LumA: 700, LumB: 475, Her2: 224, Basal: 209, Normal: 148	20603
	WES/WGS	-	5	1678	LumA: 673, LumB: 454, Her2: 218, Basal: 198, Normal: 135	173
	CNV	GISTIC2	5	1756	LumA: 700, LumB: 475, Her2: 224, Basal: 209, Normal: 148	22544
	Meth.	Illumina 450K BeadChip	5	1272	LumA: 500, LumB: 339, Her2: 174, Basal: 144, Normal: 115	13188

### Classifiers

To benchmark the performance of AI classifiers for cancer molecular subtype classification across 153 different benchmark datasets, we have utilized 35 distinct ML (15) and DL (20) classifiers. These classifiers encompass tree and distance-based, and advanced neural network classifiers. The selected classifiers ensure broad algorithmic diversity and representation of all major supervised learning paradigms while remaining computationally feasible across 153 datasets. The chosen models, reflecting the most commonly used methods in recent cancer subtype and multi-omics studies, enable comparability with current biomedical AI literature^87^.

Naive Bayes (NB) is a probabilistic model that uses Bayes’ theorem to assign class labels based on the conditional probabilities of features given the class label. It assumes independence among features and selects the class with the highest posterior probability^88,89^.

Tree-based models include DT^51^, RF^52,53^, and ensemble-based methods like GB^54^, HGB^55^, and AB^56^. The core working principle of tree-based models revolves around recursively splitting the dataset into subsets based on feature thresholds that maximize a splitting criterion, such as information gain or Gini impurity i.e., DT. RF introduces the concept of an ensemble of DTs, where multiple trees are trained on bootstrapped samples, and their predictions are aggregated, typically using majority voting for classification. Boosting methods like GB, HGB, and AB make small modifications to this principle. These algorithms iteratively train weak learners, often decision stumps, on datasets where misclassified samples are given higher weights. HGB optimizes this process by grouping continuous features into histograms for faster computation. In contrast, AB explicitly adjusts sample weights after each iteration, increasing the importance of misclassified samples and reducing the weight of correctly classified ones.

K-Nearest Neighbors (KNN)^59^ assigns class labels by identifying the majority class among the k-nearest neighbors of a given data point based on distance metric such as Euclidean distance. SVM^60,90^ find optimal hyperplanes in the feature space to separate classes by maximizing the margin between them. For complex, non-linear boundaries, SVM uses kernel functions to map data to higher-dimensional spaces where the separation is more straightforward.

DL classifiers encompass Multi-Layer Perceptrons (MLPs)^91^, CNNs^92^, Dense Networks (DenseNets)^93^, Residual Networks (ResNets), RNNs^94^, LSTMs^95^, Gated Recurrent Units (GRUs)^96^, and DeepGene transformer^27^. MLPs are feed-forward networks that process input features through multiple layers of interconnected neurons. Each layer applies weighted transformations followed by activation functions, with weights adjusted using backpropagation to minimize prediction error.

CNNs identify patterns by applying convolutional layers that use filters to extract features and pooling layers that reduce dimensionality while retaining critical information. ResNets build upon the CNN architecture by introducing skip connections, which directly connect input and output layers of residual blocks. Such connections enable gradients to bypass one or more layers, alleviating the vanishing gradient problem commonly encountered in deep networks. ResNets facilitate the convergence of more complex architectures, like ResNet18, ResNet34, and ResNet50, thereby improving the capacity to identify nuanced and detailed patterns in datasets. DenseNets take a complementary approach by connecting each layer to every subsequent layer within a dense block. These connections allow features learned by earlier layers to be reused throughout the network and have improved feature propagation. Variants such as DenseNet121, DenseNet161, and DenseNet169 extend the model’s capacity to learn complex interactions with fewer parameters compared to traditional deep networks.

RNNs are designed to process sequential data by incorporating loops in their architecture, enabling them to maintain a “memory” of previous inputs. However, traditional RNNs struggle with long-term dependencies due to the vanishing gradient problem. Long Short-Term Memory networks (LSTMs) overcome this limitation by introducing gates (input, forget, and output gates) to regulate the flow of information, allowing them to effectively capture long-term dependencies within sequences. GRUs simplify the LSTM architecture by combining the input and forget gates into a single update gate, reducing computational complexity while maintaining the ability to model sequential dependencies effectively. Hybrid models, such as CNN-LSTM and CNN-GRU, combine convolutional layers with recurrent layers, enabling these architectures to learn both spatial and sequential patterns in structured data.

The DeepGene transformer^27^ architecture builds upon the Transformer encoder model, integrating a multi-head self-attention mechanism with 1D convolutional layers. This hybrid design is specifically optimized for high-dimensional gene expression datasets. The model leverages the self-attention mechanism to capture long-range dependencies and intricate patterns across gene features while using 1D convolutional layers to extract local spatial patterns. This combination enables the model to prioritize biologically meaningful features efficiently without requiring prior feature selection and addresses a common limitation in conventional ML approaches.

### Evaluation measures

Following the evaluation criteria of existing cancer classification studies^97–99^, we evaluate 35 AI classifiers with 4 distinct evaluation measures namely, MACC, precision (PR), recall (RC), and F1-score (F1). Each measure is computed using a macro-averaging approach to ensure equal weighting for all cancer molecular subtypes irrespective of their sample sizes.

MACC is calculated as the average of individual accuracy scores across all cancer molecular subtypes. For a single subtype, accuracy is computed as the ratio of correctly predicted samples to the total samples in that subtype. Macro PR is calculated as the average of the ratio of true positives to total predicted positives for each subtype, with single-subtype PR computed as true positives divided by predicted positives. Macro RC is the average of the ratio of true positives to total actual positives, with single-subtype RC calculated as true positives divided by actual positives. Macro F1-score is the harmonic mean of macro-averaged PR and RC, calculated across all subtypes. For individual subtypes, the F1-score is computed as the harmonic mean of the subtype’s PR and RE.

1 $$\begin{aligned} f(x) = {\left\{ \begin{array}{ll} \text {Macro-Accuracy (MACC)} = \frac{1}{n} \sum _{i=1}^{n} \frac{T_i^+ + T_i^-}{T_i^+ + T_i^- + F_i^+ + F_i^-} \\[5pt] \text {Macro-Precision (PR)} = \frac{1}{n} \sum _{i=1}^{n} \frac{T_i^+}{T_i^+ + F_i^+} \\[5pt] \text {Macro-Recall (RC)} = \frac{1}{n} \sum _{i=1}^{n} \frac{T_i^+}{T_i^+ + F_i^-} \\[5pt] \text {Macro-F1-Score (F1)} = \frac{1}{n} \sum _{i=1}^{n} \frac{2 \cdot (PR_i \cdot RC_i)}{PR_i + RC_i} \\[5pt] \\[5pt] \end{array}\right. } \end{aligned}$$

Here, $T_i^+$ and $T_i^-$ denote the true positive and true negative predictions for subtype $i$, while $F_i^+$ and $F_i^-$ represent false positive and false negative predictions, respectively.

### Experimental setup

To preprocess benchmark datasets, we utilize two different APIs namely, TCGABioLinks (https://bioconductor.org/packages/release/bioc/html/TCGAbiolinks.html) and Datatable (https://datatable.readthedocs.io/en/latest/). Following the evaluation criteria of existing cancer molecular subtype classifiers, we perform experimentation in two different settings namely, 5-fold cross-validation and independent test. In an independent test, 80/20 data-splitting strategy is employed, where 80% of the data is used for training and 20% for testing. Validation data is split from the training data in an 80:20 ratio prior to classifier training. In addition, stratification is applied to ensure that each class is proportionally represented in both the training and test sets^100^. 5-fold cross-validation is also performed with stratification to provide a more comprehensive evaluation of model performance and reduce potential biases introduced by specific data splits^101^. All of the ML classifiers are developed on top of Scikit-Learn v1.3.2 (https://scikit-learn.org/stable). The DL classifiers are developed with Pytorch (https://pytorch.org/). All visualizations are generated using matplotlib v3.8.0 (https://matplotlib.org/) and PlotNine (https://plotnine.org/).

**Fig. 2:**
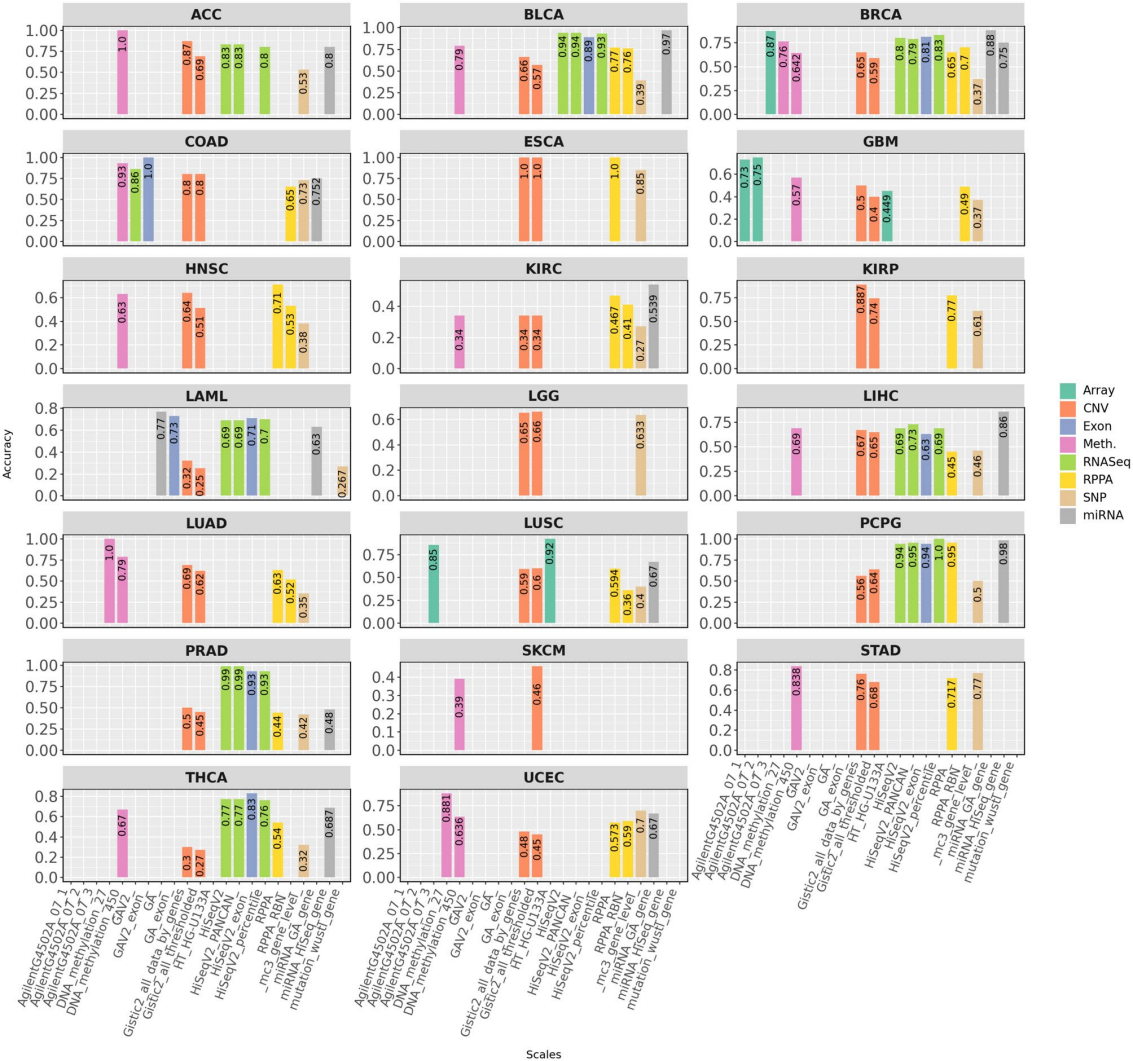
MACC values of top performing classifiers in terms of 20 different configurations of 8 distinct omics modalities across 20 different cancers.

## Results

This section layer by layer unfolds the predictive performance of 35 unique ML and DL classifiers across 153 datasets of 8 distinct omics modalities in terms of 20 different cancers. First, it presents a performance comparison of AI classifiers on 17 unique configurations of 5 out of 8 tested omics modalities to identify the most effective configurations for each omics modality. Next, it unveils the potential of 8 distinct omics modalities for cancer molecular subtype classification across 20 unique cancers. In addition, it also discusses the performance trends of 35 ML and DL classifiers for 8 omics modalities and 20 different cancers. Finally, it furnishes information about top-performing configuration, modality, and classifier combinations and provides insights into the strengths and weaknesses of ML and DL classifiers for cancer molecular subtype classification. It is important to note that the results presented in this section are derived from independent testing, while 5-fold cross-validation results are presented in Supplementary File 2.

### RQ I: What data configurations are critical for accurate cancer molecular subtype classification?

As TCGA provides multiple dataset configurations for different omics modalities as described in section “Datasets” and Table 1, selecting the optimal configuration plays a key role in cancer molecular subtype classification. To illustrate this, this subsection answers RQ I by evaluating the classification performance of 17 dataset configurations from 5 omics modalities (Array, CNV, Meth., RNASeq, and RPPA), due to dataset availability constraints. By assessing ML and DL classifiers on independent test sets across 20 cancers, this study determines the most effective dataset configurations based on MACC scores, which offers insights into their suitability for cancer molecular subtype classification.

Figure 2 illustrates the highest MACC achieved by ML and DL classifiers across 17 configurations of 5 out of 8 distinct omics modalities, whereas detailed performance scores are provided in Supplementary File 1 Table S2. For CNV, classifiers trained on Gistic2-all-data-genes achieve a higher average MACC of 0.624, outperforming Gistic2-all-thresholded, which achieves 0.568. This trend is particularly evident from the calculated differences in MACC scores across 13 cancers, including ACC (0.18), BLCA (0.09), BRCA (0.06), GBM (0.1), HNSC (0.13), KIRP (0.147), LAML (0.07), LIHC (0.02), LUAD (0.07), PRAD (0.05), STAD (0.08), THCA (0.03), and UCEC (0.03). In contrast, Gistic2-all-thresholded demonstrates either superior or equivalent performance in 6 cancers: COAD, ESCA, KIRP, LGG (0.01), LUSC (0.01), and PCPG (0.08), where the values represent the calculated differences in MACC scores between the two datasets. Notably, SKCM is excluded from this comparison due to the absence of a Gistic2-all-thresholded dataset. Classifiers trained with Gistic2-all-data-genes achieve better predictive performance due to its granular, continuous representation of gene-level copy number variations, which preserves subtle patterns and rich genomic details critical for cancer molecular subtype classification. Conversely, Gistic2-all-thresholded simplifies data into discrete categories (gain, loss, neutral), effectively reducing noise but potentially discarding critical genomic variability.

In terms of RNASeq, four configurations, namely HiSeqV2, HiSeqV2-PANCAN, HiSeqV2-percentile, and GAV2—are considered. However, GAV2 is excluded from this analysis due to insufficient data across multiple cancers. A closer examination of MACC reveals that classifiers trained on HiSeqV2, HiSeqV2-PANCAN, and HiSeqV2-percentile exhibit nearly identical performance, achieving average MACC scores of 0.83, 0.84, and 0.83, respectively, across 8 cancers: ACC, BLCA, BRCA, LAML, LIHC, PCPG, PRAD, and THCA. Notably, COAD, ESCA, GBM, HNSC, KIRC, KIRP, LGG, LUAD, LUSC, SKCM, STAD, and UCEC are excluded from this analysis due to a lack of comparable data (uniformly processed, subtype-annotated RNA-Seq datasets compatible with the HiSeqV2-based benchmarking framework). The consistent performance across these RNASeq configurations suggests that they preserve key biological signals while minimizing noise, ensuring robustness in cancer molecular subtype classification regardless of the preprocessing or normalization strategy.

**Fig. 3:**
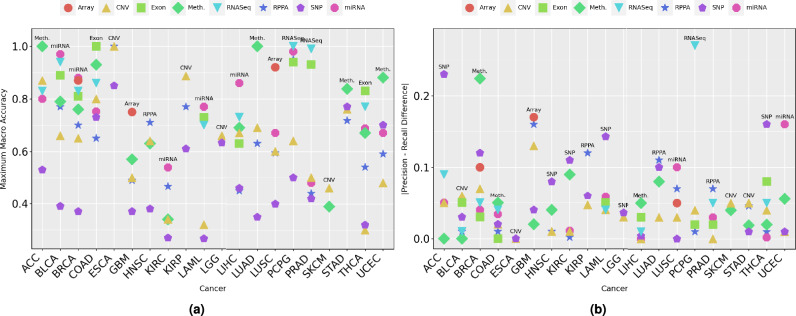
(**a**) Macro-accuracy of top performing classifier-modality combination across 20 cancers. (**b**) PR–RC difference of top performing classifier-modality combination for each cancer. Larger PR–RC difference indicates higher bias in the subtype classification results.

In the context of protein expression, a comparison between RPPA and RPPA-RBN across seven cancers shows that classifiers trained on RPPA achieve a higher average MACC (0.627) compared to RPPA-RBN (0.552). Specifically, RPPA outperforms RPPA-RBN in five cancers: BLCA (0.01), HNSC (0.18), LUSC (0.23), KIRC (0.057), and LUAD (0.11). In contrast, RPPA-RBN slightly outperforms RPPA in BRCA (0.05) and UCEC (0.017). The calculated differences highlight the superior performance of RPPA over RPPA-RBN in most cancers. This may be attributed to its ability to preserve critical protein expression signals without additional transformations. By avoiding normalization, RPPA better captures unique molecular profiles and protein expression variations across different cancers, whereas RPPA-RBN’s normalization process may obscure subtle subtype-specific differences.

For the array modality, 4 configurations (AgilentG4502A-07-1, AgilentG4502A-07-2, AgilentG4502A-07-3, and HTHG-U133A) are evaluated across GBM (AgilentG4502A-07-1 and AgilentG4502A-07-2) and LUSC (AgilentG4502A-07-3 and HTHG-U133A). While this analysis does not encompass all configurations across cancers, it provides valuable insights into their performance in specific cases. For GBM, classifiers trained on AgilentG4502A-07-2 slightly outperform those trained on AgilentG4502A-07-1, with a MACC difference of 0.02. This minor out-performance may be attributed to more refined data preprocessing in AgilentG4502A-07-2, potentially better aligning with the molecular characteristics of GBM. However, further comparisons across additional cancers and configurations are needed for conclusive insights. For LUSC, classifiers trained on HTHG-U133A outperform those using AgilentG4502A-07-3 by a margin of 0.07. Although this difference is modest, it may be due to HTHG-U133A offering a broader dynamic range or improved feature representation, enhancing the detection of subtype-specific patterns in LUSC. Nonetheless, additional experiments and datasets are required to validate these findings across broader conditions.

For Meth. modality, the analysis is conducted only on BRCA and LUAD, as the two available configurations ie., HumanMethylation27 (HM27) and HumanMethylation450 (HM450) are limited to these cancers. In BRCA, classifiers trained on HM27 outperform those using HM450, with a MACC margin of 0.118. Similarly, in LUAD, HM27-based classifiers demonstrate superior performance with a MACC margin of 0.21. Classifiers trained on HM27 outperform those on HM450 due to a higher signal-to-noise ratio, as HM27 focuses on promoter CpG islands, while HM450 includes many low-variance, sparsely methylated sites that add noise. The lower dimensionality of HM27 ( 27k vs.  450k features) helps avoid overfitting, whereas HM450’s high feature-to-sample ratio and batch effects reduce classifier robustness.

**Fig. 4:**
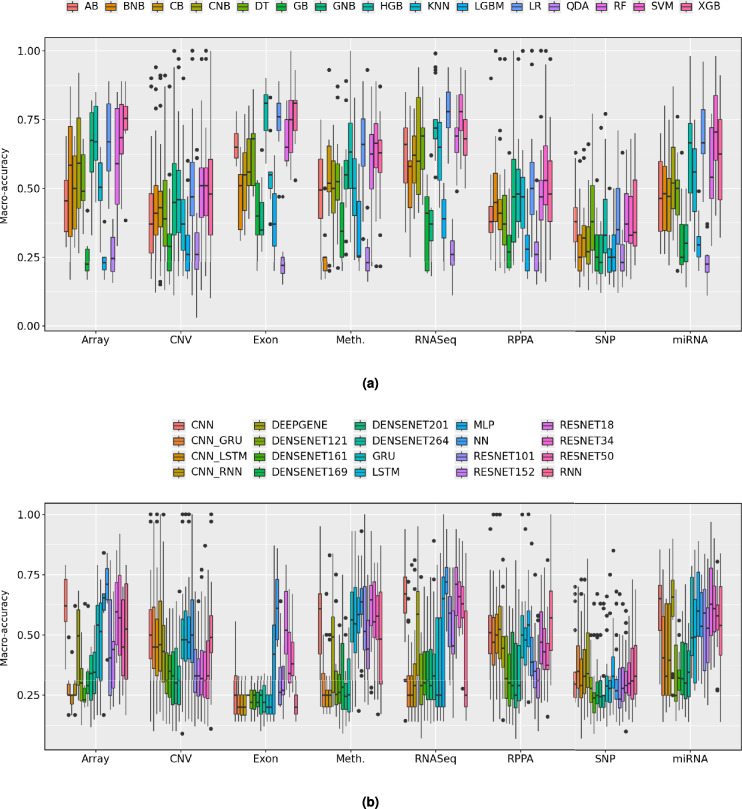
Modality-wise average MACC values for (**a**) ML, (**b**) DL classifiers. For each omics modality, the average performance of a classifier is computed by aggregating its MACC values across all cancers. It highlights the comparative effectiveness of different classifiers w.r.t modalities for cancer molecular subtype classification.

Overall, the analyses of 17 dataset configurations across 5 distinct omics modalities conclude that Gistic2-all-data-genes outperforms Gistic2-all-thresholded for cancer molecular subtype classification in CNV. In RNASeq, HiSeqV2, HiSeqV2-PANCAN, and HiSeqV2-percentile achieve nearly identical performance, which confirms their robustness across cancers. In RPPA, unprocessed data provides better results than RPPA-RBN, as it retains essential protein expression signals. The array modality shows variations based on configuration, while in Meth., HM27 outperforms HM450 due to a higher signal-to-noise ratio and a lower risk of overfitting. These results emphasize the importance of choosing appropriate dataset configurations, as differences in preprocessing and representation methods impact predictive performance of classifiers for cancer molecular subtype classification.

### RQ II & III) How consistently do different omics modalities perform in cancer subtype classification across diverse cancers?

In the previous subsection, we determined the most effective configurations for each omics modality for cancer molecular subtype classification. Building on that analyses, this subsection evaluates the effectiveness of 8 distinct omics modalities (Array, CNV, Meth., RNASeq, RPPA, SNP, Exon, and miRNA) in cancer molecular subtype classification across 20 different cancers. To illustrate this, this subsection answers RQ II and III by presenting the maximum MACC and minimum PR-RE disparity achieved by an ML or DL classifier for each of the 8 omics modalities. This analysis aims to highlight the strengths and limitations of each omics modality in achieving accurate and robust cancer molecular subtype classification. In addition, the availability of 8 omics modalities is not uniform across all cancers, hence the analyses and conclusions are drawn based on the omics modalities present for each cancer.

Figure 3a illustrates the maximum MACC achieved by the top-performing classifiers for 20 different cancers across 8 omics modalities: Array, CNV, RNASeq, RPPA, SNP, Exon, Meth., and miRNA, with a detailed breakdown of these performance scores provided in Supplementary File 1 Table S2. The performance analysis of omics modalities across 20 different cancers highlights distinct trends i.e., omics modalities with maximum and minimum performances, disparity of PR and RE across omics modalities. Classifiers trained with specific omics modalities achieving maximum performance demonstrate the effectiveness of these modalities for cancer molecular subtype classification, and vice versa. For instance, classifiers trained on miRNA modality perform better in terms of MACC across 5 out of 12 cancers (BLCA, BRCA, KIRC, LAML, and LIHC). This trend establishes miRNA as the most informative omics modality overall for cancer molecular subtype classification. Similarly, classifiers trained on Meth. modality achieve maximum performance in 4 out of 13 cancers namely, ACC, LUAD, UCEC, and STAD. Similarly, classifiers trained with CNV modality demonstrate better MACC in 4 out of 20 cancers (KIRP, ESCA, SKCM, and KIRP). In addition, classifiers based on Exon modality show better performance in 2 out of 8 cancers namely, COAD, and THCA. Although the RNASeq modality allows classifiers to achieve the highest MACC 2 out of 9 cancers (PCPG, and PRAD), these classifiers consistently demonstrate reasonable performance compared to those based on other modalities. Classifiers using Array and RPPA modalities perform best in 3 cancers (Array (2 out of 3): GBM, LUSC; RPPA (1 out of 14): HNSC). Conversely, classifiers trained on SNP modality frequently show the lowest performance, particularly in cancers such as ACC, LAML, RPPA, BLCA, BRCA, KIRP, GBM, KIRC, HNSC, KIRP, LAML, LGG, LUAD, LUSC, PCPG, PRAD, and THCA. This indicates the reduced predictive capacity for RPPA modality in 12 cancers for cancer molecular subtype classification.

Figure 3b shows the disparity between macro PR and RE across 20 cancers and 8 omics modalities. This disparity reflects that PR does not always align with RE, which highlights potential biases or limitations in cancer molecular subtype classification. Classifiers using SNP modality exhibit the highest disparity in 5 cancers, such as ACC (0.23), KIRC (0.11), LAML (0.14), HNSC (0.08) and TCHA (0.16). This highlights challenges in achieving accurate molecular subtype classification for these cancers, even though SNP-based classifiers may achieve moderate PR in some cases. Similarly, classifiers using RPPA modality encounter notable disparity in 3 cancers like KIRP (0.12), PRAD (0.07), and LUAD (0.11), emphasizing the need for optimization in leveraging RPPA data for consistent cancer molecular subtype classification. While certain modality-classifier combinations may achieve high MACC in classification tasks, this does not necessarily imply the absence of bias. For instance, classifiers trained on Array (GBM: 0.17), miRNA (UCEC: 0.16, LUSC: 0.1), and RNASeq (PCPG: 0.27) exhibit significant PR-RE disparity. These findings highlight that, despite strong overall performance in some cases, the presence of disparity must be carefully considered when using these modalities for cancer molecular subtype classification.

Certain modalities exhibit better alignment between macro PR and RE, achieving more consistent performance across cancers. RNASeq-based classifiers demonstrate low disparity in cancers such as ACC (0.09), LAML (0.04), and BLCA (0.01), showcasing their strength in maintaining balanced PR and RE for cancer classification. Similarly, classifiers trained on the CNV modality exhibit consistently low disparity across 19 cancers, although a slightly higher disparity is observed in GBM (0.13). This suggests that CNV-based classifiers are generally robust but may encounter challenges in achieving consistent classification for GBM. Moreover, miRNA-based classifiers show low disparity in diverse cancers such as PCPG, PRAD, COAD, and BRCA, demonstrating their robustness and stability. Additionally, classifiers trained on Exon modality maintain consistent performance with relatively low disparity in cancers such as COAD (0), and PRAD (0.02). These findings underline the potential of RNASeq, miRNA, Exon, and CNV data for achieving reliable and consistent molecular subtype classification across cancers. In summary, while modalities like RNASeq, miRNA, Exon, and CNV demonstrate consistent performance, challenges faced by classifiers using SNP, RPPA, and Array data highlight the need for further optimization and targeted strategies to improve their effectiveness in cancer molecular classification tasks.

In summary, classifiers trained on miRNA, RNASeq, Exon, Meth., and CNV data emerge as the most effective for cancer molecular subtype classification. miRNA-based classifiers achieve top performance in 5 cancers (BLCA, BRCA, KIRC, LAML, and LIHC), while CNV-based classifiers perform best in 4 cancers (KIRP, ESCA, SKCM, and KIRP). Classifiers utilizing RNASeq and Exon data also demonstrate strong performance, achieving top accuracy in 2 cancers each (RNASeq: PCPG, PRAD; Exon: COAD, THCA). These modalities consistently exhibit low disparity between precision and recall across various cancers, such as ACC, BLCA, and THCA, ensuring more balanced classification outcomes. In contrast, classifiers trained on SNP and RPPA data often show higher disparities and lower predictive performance, particularly in cancers such as ACC, LAML, and HNSC. While CNV-based classifiers are generally robust, a slightly higher disparity observed in GBM suggests potential challenges in maintaining consistent classification performance for this cancer. Similarly, classifiers using RPPA data encounter notable disparities in cancers like KIRP, LUSC, and LUAD, indicating the need for further optimization. These findings emphasize the importance of selecting data modalities based on their predictive capacity for specific cancers and addressing modality-specific biases to improve molecular subtype classification.

### RQ IV)a Which ML classifiers demonstrate reliable performance across all omics modalities?

In the previous subsection, we identified the omics data modalities that provide the most effective and accurate cancer molecular subtype classification by analyzing the maximum MACC and PR-RE disparity of ML and DL classifiers. However, it is equally important to assess how individual ML classifiers perform across each omics modality. To address this, we investigate RQ IVa by conducting an in-depth analysis of the median and inter-quartile range (IQR) of the MACC of 15 different ML classifiers across 8 distinct omics modalities for 20 cancers. Classifiers are categorized into three groups based on the median and interquartile range (IQR) of their MACC: high-performing classifiers (high MACC and low IQR, indicating robustness and consistency), moderate-performing classifiers (high MACC but high IQR, variability despite strong performance), and low-performing classifiers (low MACC and high IQR, inconsistency and reduced reliability). For each omics modality, the median MACC values of all classifiers are computed and ranked in descending order. Classifiers within the top one-third of the modality-specific performance range designated as high-performing, those in the middle third as moderate-performing, and those in the lowest third as low-performing. These insights guide the selection of robust classifiers tailored to omics modalities for cancer molecular subtype classification.

Figure 4a and Supplementary Table 4 illustrate median MACC and IQR of 15 distinct ML classifiers in terms of 8 different omics modalities across 20 different cancers, with detailed performance scores presented in Supplementary File 1 Table S3. On the basis of median MACC and IQR, 3 different performance groups are derived which are presented in Table 3. In the Array modality, XGB exhibits superior predictive performance, achieving a median MACC of 0.755 and an IQR of 0.085, indicating strong performance alongside low variability. SVM, GNB, and HGB also show competitive results, with median MACC values of 0.685, 0.675, and 0.670, respectively, though they exhibit higher variability (IQRs: 0.180, 0.215, and 0.195). LR follows closely with MACC of 0.670, with varying levels of consistency. Among the moderate performers, CNB and RF show a decline in performance, with MACC values of 0.592 and 0.590, and higher variability (IQRs: 0.328 and 0.347) showing inconsistencies across cancer subtypes. BNB and KNN perform even lower, with MACC values of 0.585 and 0.505, and high disparity in performance across different cases. The lowest-performing classifiers in the Array modality include CB, DT, and AB, with MACC values of 0.500, 0.490, and 0.455, respectively. QDA, LGBM, and GB show the lowest results, with median MACC values dropping as low as 0.245, 0.230, and 0.225, respectively, accompanied by low IQR values, which suggest instability in their predictions. These findings indicate that XGB, SVM, and GNB are among the most effective classifiers for the Array modality in cancer molecular subtype classification, while QDA, LGB, and GB struggle to provide reliable results.

**Table 3:** Three different categories of ML classifiers are assigned i.e., $\bigstar$ denotes classifiers with top performance for a modality, $\bullet$ shows the moderate performing classifiers for a modality and $\times$ represents the lowest performance.

Classifier	Array	CNV	Exon	Meth.	RNASeq	RPPA	SNP	miRNA
AB	$\times$	$\times$	$\times$	$\times$	$\bullet$	$\times$	$\times$	$\times$
BNB	$\bullet$	$\times$	$\times$	$\times$	$\times$	$\times$	$\times$	$\times$
CB	$\times$	$\times$	$\bullet$	$\times$	$\bullet$	$\times$	$\times$	$\times$
CNB	$\bullet$	-	$\bullet$	$\times$	$\bullet$	-	$\times$	$\times$
DT	$\times$	$\times$	$\times$	$\bullet$	$\bigstar$	$\times$	$\times$	$\times$
GNB	$\bigstar$	$\times$	$\times$	$\bullet$	$\times$	$\times$	$\times$	$\times$
GB	$\times$	$\times$	$\times$	$\times$	$\times$	$\times$	$\times$	$\times$
HGB	$\bigstar$	$\times$	$\bigstar$	$\bigstar$	$\bigstar$	$\bullet$	$\times$	$\bigstar$
KNN	$\bullet$	$\times$	$\bullet$	$\times$	$\bullet$	$\bullet$	$\times$	$\bullet$
LGBM	$\times$	$\times$	$\times$	$\times$	$\times$	$\times$	$\times$	$\times$
LR	$\bigstar$	$\bullet$	$\bigstar$	$\bigstar$	$\bigstar$	$\bigstar$	$\times$	$\bigstar$
QDA	$\times$	$\times$	$\times$	$\times$	$\times$	$\times$	$\times$	$\times$
RF	$\bullet$	$\bigstar$	$\times$	$\bigstar$	$\bigstar$	$\bullet$	$\times$	$\times$
SVM	$\bigstar$	$\bigstar$	$\times$	$\bigstar$	$\bigstar$	$\bigstar$	$\times$	$\bigstar$
XGB	$\bigstar$	$\bullet$	$\bigstar$	$\times$	$\bigstar$	$\bullet$	$\times$	$\bullet$

In the CNV modality, RF and SVM achieve the highest median MACC of 0.51 with IQRs of 0.180 and 0.175. XGB, and LR follow with MACC values of 0.48, and 0.47, and IQRs of 0.275, and 0.195. HGB and GNB perform moderately with MACC values of 0.46 and 0.45 and IQRs of 0.225 and 0.260. CB and BNB show slightly lower performance with median MACC values of 0.43 and 0.41 and IQRs of 0.130 and 0.180. DT, AdaBoost, and KNN perform worse with MACC values of 0.39, 0.37, and 0.37 and IQRs of 0.240, 0.215, and 0.185. GB, LGBM, and QDA show the lowest performance with MACC values of 0.29, 0.26, and 0.26 and moderate IQR values. RF, SVM, and XGB perform best in the CNV modality. QDA, LGBM, and GB show the lowest results. CNB is not applicable to CNV data due to negative values across all configurations of CNV modality.

For the SNP modality, none of the classifiers show reasonable performance, as all classifiers achieve low median MACC values with high variability. AB and DT perform the best but only achieve 0.38 MACC with inconsistent results (IQR: 0.125 and 0.235). RF, LR, XGB, and HGB fall within the 0.33–0.37 range, but all exhibit high variability. The remaining classifiers, including SVM, CB, CNB, BNB, GB, KNN, LBGM, QDA, and GNB, perform even worse, with MACC values dropping to 0.230–0.330.

The RNASeq modality features LR and SVM as the top performers, both achieving a MACC of 0.78 and a moderate IQR of 0.130. HGB follows closely, with a MACC of 0.72 and a lower IQR of 0.070. DT and RF perform moderately, reaching a MACC of 0.69 and IQRs of 0.150 and 0.090, respectively. XGB achieves a 0.68 MACC with an IQR of 0.130, making it another competitive choice. AB, and KNN also show moderate performance, with MACC values between 0.66 and 0.65. CB and CNB perform moderately, with MACC values of 0.62 and 0.60 and IQRs of 0.170 and 0.352. The weakest classifiers, BNB, GB, LGBM, GNB, and QDA, achieve MACC values between 0.58 and 0.26, confirming their ineffectiveness for RNASeq-based cancer molecular subtype classification.

In the Exon modality, HGB and XGB achieve the highest performance, both with a MACC of 0.81 and IQRs of 0.13 and 0.11. LR follows closely with a MACC of 0.76 and a low IQR of 0.10. SVM performs well with a MACC of 0.75 but shows higher variability (IQR: 0.19). Among the moderate performers, DT, AB, and RF show MACC values of 0.68, 0.65, and 0.65, with IQRs between 0.09 and 0.19, demonstrating mixed levels of consistency. CNB, CB, and KNN achieve lower performance, with MACC values of 0.56 and 0.55, but KNN has the lowest IQR (0.06), indicating more stable predictions. The weakest classifiers include BNB, GB, LGBM, GNB, and QDA, all with MACC values below 0.51. QDA exhibits the worst performance, with a MACC of 0.22 and an IQR of 0.06, which shows its ineffectiveness for Exon-based cancer molecular subtype classification.

In the Meth. modality, LR and SVM perform the best, both achieving a MACC of 0.67 with moderate variability (IQR: 0.1725 and 0.1600). RF (0.62), HGB (0.633), and XGB (0.630) follow closely. GNB (0.55), and DT (0.525) perform moderately but with increased variability. CB, CNB, and KNN fall below 0.52, indicating weaker predictive capability. The lowest-performing classifiers include GB (0.34), LGBM (0.26), BNB (0.25), and QDA (0.23), confirming their ineffectiveness for Meth.-based classification.

In the RPPA modality, none of the classifiers perform well, with all showing low median MACC and high variability. SVM (0.53), and LR (0.50) achieve the highest scores but remain suboptimal. HGB, XGB, RF, and KNN perform similarly (0.48–0.47) with inconsistent results, especially GNB (IQR: 0.300). BNB, CatBoost, AdaBoost, DT, LGBM, GB, and QDA also fall below 0.46 which suggests the ineffectiveness for the use of ML classifiers for cancer molecular subtype classification.

In the miRNA modality, SVM (0.705) shows the best performance but with high variability (IQR: 0.235). HGB and LR (0.665) follow, with HGB exhibiting greater inconsistency (IQR: 0.25) than LR (0.16). XGB (0.625) performs moderately, though XGB shows high instability (IQR: 0.292). Lower performers include KNN (0.56), RF (0.54), CNB (0.52), and DT (0.50) while BNB, CB, and AB (0.48–0.46) demonstrate weaker predictive capacity. The worst classifiers, GNB, LightGBM, GBoost, and QDA ($\le$ 0.30), show their limited ability to handle miRNA modality for cancer molecular subtype classification.

Out of 16 different ML classifiers, only 6 i.e., LR, SVM, HGB, XGB, and RF consistently demonstrate high median MACC values with relatively low IQRs. Their strong predictive capability makes them the most suitable choices for cancer molecular subtype classification. In contrast, BNB, AB, CB, DT, GNB, GB, KNN, LGBM, MLP, and QDA demonstrate the lowest performance across all omics modalities. Their poor accuracy and high variability limit their effectiveness for cancer molecular subtype classification. These trends highlight the importance of balancing median MACC and IQR when selecting classifiers for specific modalities.

**Fig. 5:**
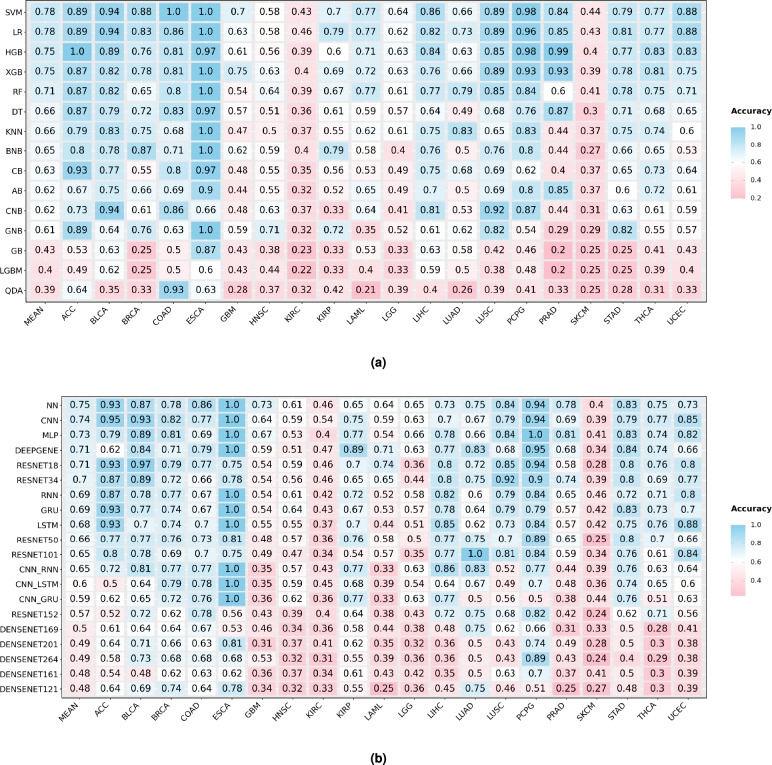
Across each cancer, the top performance of a classifier is shown regardless of the omics modality or dataset configuration to identify the most consistent classifiers for cancer molecular subtype classification.

### RQ IV)b What DL classifiers demonstrate reliable performance across all omics modalities?

Following the insights of ML classifiers from the previous subsection, hereby we address RQ IVb to assess the performance of 20 different DL classifiers across 8 distinct omics modalities in terms of 20 different cancers based on independent testing. The grouping of classifiers into three categories such as high-performing (high MACC and low IQR), moderate-performing (high MACC but high IQR), and low-performing (low MACC and high IQR) is done as explained in the previous subsection. This classification provides insights into the reliability of different DL models for cancer molecular subtype classification across omics modalities.

**Table 4:** Three different categories of DL classifiers i.e., $\bigstar$ denotes classifiers with top performance for a modality, $\bullet$ shows the moderate performing classifiers for a modality and $\times$ represents the lowest performance.

Classifier	Array	CNV	Exon	Meth.	RNA	RPPA	SNP	miRNA
MLP	$\bullet$	$\bullet$	$\times$	$\bullet$	$\bullet$	$\bigstar$	$\times$	$\bigstar$
NN	$\bigstar$	$\bigstar$	$\bigstar$	$\bigstar$	$\bigstar$	$\bigstar$	$\times$	$\bigstar$
CNN	$\bigstar$	$\bigstar$	$\times$	$\bullet$	$\bullet$	$\bigstar$	$\times$	$\bigstar$
ResNet18	$\bullet$	$\times$	$\bigstar$	$\bigstar$	$\bigstar$	$\times$	$\times$	$\bigstar$
ResNet34	$\bullet$	$\times$	$\times$	$\bullet$	$\bullet$	$\times$	$\times$	$\bullet$
GRU	$\bullet$	$\bullet$	$\times$	$\bullet$	$\times$	$\bigstar$	$\times$	$\bullet$
RNN	$\times$	$\bullet$	$\times$	$\times$	$\times$	$\bigstar$	$\times$	$\bullet$
LSTM	$\times$	$\bullet$	$\times$	$\times$	$\times$	$\bullet$	$\times$	$\bullet$
DEEPGEN	$\times$	$\times$	$\times$	$\times$	$\times$	$\bullet$	$\times$	$\bigstar$
ResNet50	$\times$	$\times$	$\bullet$	$\bullet$	$\bullet$	$\times$	$\times$	$\times$
ResNet101	$\times$	$\times$	$\times$	$\times$	$\times$	$\times$	$\times$	$\times$
ResNet152	$\times$	$\times$	$\times$	$\times$	$\times$	$\times$	$\times$	$\times$
DenseNet101	$\times$	$\times$	$\times$	$\times$	$\times$	$\times$	$\times$	$\times$
DenseNet201	$\times$	$\times$	$\times$	$\times$	$\times$	$\times$	$\times$	$\times$
DenseNet121	$\times$	$\times$	$\times$	$\times$	$\times$	$\times$	$\times$	$\times$
DenseNet169	$\times$	$\times$	$\times$	$\times$	$\times$	$\times$	$\times$	$\times$
CNN-GRU	$\times$	$\times$	$\times$	$\times$	$\times$	$\bullet$	$\times$	$\times$
CNN-RNN	$\times$	$\times$	$\times$	$\times$	$\times$	$\bigstar$	$\times$	$\bullet$
CNN-LSTM	$\times$	$\times$	$\times$	$\times$	$\times$	$\bullet$	$\times$	$\times$
DenseNet161	$\times$	$\times$	$\times$	$\times$	$\times$	$\times$	$\times$	$\times$

Figure 4b illustrates median MACC and IQR of 20 distinct DL classifiers across 8 different omics modalities in terms of 20 different cancers, with detailed performance scores presented in Supplementary File 1 Table S5. On the basis of median MACC and IQR, 3 different performance groups are derived which are presented in Table 4. In the Array modality, NN (0.71, IQR: 0.13) shows the best performance, followed by MLP (0.655, IQR: 0.067) and CNN (0.62, IQR: 0.175). ResNet18 (0.595) ResNet34 (0.57), and GRU (0.54) perform well but with high variability (IQR: 0.26–0.30). The lowest-performing classifiers include ResNet101, DenseNet models (264, 201, 121, 169), RNN, LSTM, DeepGene Transformer, Resnet50, CNN-GRU, CNN-RNN, CNN-LSTM, all with MACC $\le$ 0.525, which proves their ineffectiveness for Array-based cancer molecular subtype classification.

In the CNV modality, NN and CNN achieve the highest performance with a median MACC of 0.50, though CNN shows lower variability (IQR: 0.205) compared to NN (IQR: 0.260). GRU (0.48), RNN (0.49), LSTM (0.48), and MLP (0.47) fall into the moderate-performing category, with relatively moderate variability (IQR: 0.155–0.210). The lowest-performing classifiers include CNN-RNN, CNN-GRU, CNN-LSTM, DeepGene transformer, ResNet (18, 34, 50, 101, 152), and DenseNet (121, 161, 169, 201, 264), all with MACC $\le$ 0.46, which prove their limited reliability for CNV-based cancer molecular subtype classification.

In the Exon modality, NN achieves the highest performance with a MACC of 0.61, though its high IQR of 0.25 indicates some inconsistency. ResNet18 (0.52) and MLP (0.42) show moderate performance, but both have high IQR values of 0.26 and 0.37, which affects their stability. The lowest-performing classifiers include ResNet34, ResNet152, ResNet101, CNN, CNN-RNN, CNN-LSTM, CNN-GRU, DenseNet models (121, 161, 169, 201, 264), and recurrent models (GRU, LSTM, RNN). All have MACC values of 0.38 or lower, which confirms their poor performance in Exon-based classification.

In the Meth. modality, ResNet18 (0.65) and NN (0.64) achieve the highest performance, with low IQR values of 0.16 and 0.108, which makes them the most effective classifiers. CNN (0.609), MLP (0.585), RESNET50 (0.58), GRU (0.56), and RESNET34 (0.55) show moderate performance, but their high IQR values between 0.14 and 0.25 affect their stability. The lowest-performing classifiers include RNN, LSTM, ResNet101, ResNet152, DeepGene, DenseNet models (121, 161, 169, 201, 264), CNN-GRU, CNN-RNN, and CNN-LSTM. All have MACC values of 0.54 or lower, which confirms their poor performance in Meth.-based cancer molecular subtype classification

In the RNASeq modality, NN (0.72) and ResNet18 (0.71) achieve the highest median MACC, with IQR values of 0.1175 and 0.21, which makes them the most reliable classifiers. CNN (0.67), MLP (0.65), ResNet34 (0.66), and ResNet50 (0.63) show moderate performance, with IQR values of 0.15, 0.54, 0.19, and 0.13, which indicates some variability. The lowest-performing classifiers include ResNet101 (0.59), ResNet152 (0.454), DeepGene (0.586), DenseNet models (121: 0.29, 161: 0.30, 169: 0.31, 201: 0.29, 264: 0.33), LSTM (0.25), GRU (0.25), RNN (0.25), CNN-RNN (0.29), CNN-GRU (0.25), and CNN-LSTM (0.25). These classifiers have MACC values of 0.59 or lower, which confirms their poor performance in RNASeq-based classification.

In the RPPA modality, RNN (0.57), MLP (0.54), and CNN-RNN (0.52) achieve the highest median MACC, with IQR values ranging from 0.15-0.24. CNN-GRU (0.47), GRU (0.50), CNN (0.51), and CNN-LSTM (0.50) also perform well, with IQR values between 0.15 and 0.22. LSTM (0.479), ResNet18 (0.47), DeepGene (0.45), and ResNet34 (0.43) show moderate performance, but higher IQR values in some cases affect their consistency. The lowest-performing classifiers include DenseNet models (121: 0.32, 161: 0.30, 169: 0.29, 201: 0.25, 264: 0.29), ResNet models (50: 0.37, 101: 0.35, 152: 0.31), and NN (0.38). These classifiers have MACC values of 0.38 or lower which indicates their poor performance in RPPA-based cancer molecular subtype classification. In addition, none of the DL classifiers perform well with SNP. All DL classifiers achieve MACC values below 0.35, with high variability, confirming SNP as an ineffective modality for DL-based cancer molecular subtype classification classification. In the miRNA modality, ResNet18 (0.63), CNN (0.65), MLP (0.60), and DEEPGENE transformer (0.66) achieve the highest performance, with high variability (IQR: 0.14- 0.27). CNN-RNN, RESNET34, RNN, GRU, and LSTM also perform well with median MACC 0.39-0.59, but CNN-RNN has the highest IQR (0.37). Moderate performers include DenseNet models (0.25-0.32), ResNet152 (0.53) and ResNet101 (0.59), with IQR values of 0.22 and 0.34 and low performers include CNN-GRU (0.41) and CNN-LSTM (0.33), with IQR values above 0.35.

DL classifiers show distinct performance trends across 8 omics modalities. NN and ResNet18 consistently achieve high median MACC with low IQR, making them the most reliable classifiers across multiple modalities, including Array, CNV, Exon, Methylation, and RNASeq. CNN and MLP perform well in RNASeq, RPPA, Array, CNV, and miRNA, though with slightly higher variability. Hybrid models like CNN-RNN and CNN-GRU perform effectively in RPPA and miRNA, indicating their ability to handle complex omics structures. Moderate performers such as ResNet34, ResNet50, GRU, and LSTM exhibit reasonable median MACC but with higher variability, limiting their consistency. Low-performing classifiers, including DenseNet models, ResNet101, ResNet152, and recurrent models like RNN and LSTM, struggle across most modalities, showing poor accuracy and high IQR. SNP emerges as the least effective modality for DL classifiers, with all models failing to achieve reliable performance.

**Table 5:** Top-performing classifier-modality-configuration combinations for cancer molecular subtype classification.

Cancer	Modality	Configuration	Classifier	ACC	PR	RE	F1
ACC	Meth.	HumanMethylation450	HGB	1.000	1.000	1.000	1.000
BLCA	miRNA	miRNA-HiSeq-gene	RESNET18	0.970	0.970	0.970	0.970
BRCA	miRNA	miRNA-GA-gene	SVM	0.880	0.810	0.880	0.830
COAD	Exon	GAV2-exon	SVM	1.000	1.000	1.000	1.000
ESCA	CNV	Gistic2-all-data-by-genes	CNN	1.000	1.000	1.000	1.000
GBM	Array	AgilentG4502A-07-2	XGB	0.750	0.800	0.750	0.760
HNSC	RPPA	RPPA	GNB	0.710	0.710	0.710	0.700
KIRC	RPPA	RPPA	CNN	0.539	0.550	0.539	0.536
KIRP	CNV	Gistic2-all-data-by-genes	DEEPGENE	0.887	0.871	0.887	0.875
LAML	miRNA	GA	SVM	0.77	0.829	0.77	0.759
LGG	CNV	Gistic2-all-thresholded	MLP	0.660	0.580	0.660	0.610
LIHC	miRNA	miRNA-HiSeq-gene	SVM	0.860	0.860	0.860	0.860
LUAD	Meth.	HumanMethylation27	RESNET101	1.000	1.000	1.000	1.000
LUSC	Array	HT-HG-U133A	RESNET34	0.920	0.970	0.920	0.930
PCPG	RNASeq	HiSeqV2-percentile	MLP	1.000	1.000	1.000	1.000
PRAD	RNASeq	HiSeqV2-PANCAN	HGB	0.990	0.990	0.990	0.990
SKCM	CNV	Gistic2-all-thresholded	RNN	0.460	0.410	0.460	0.430
STAD	Meth.	HumanMethylation450	DEEPGENE	0.838	0.819	0.838	0.827
THCA	Exon	HiSeqV2-exon	HGB	0.830	0.910	0.830	0.850
UCEC	Meth.	HumanMethylation27	LSTM	0.881	0.937	0.881	0.900

### RQ V) Which specific ML and DL classifiers provide consistent predictive performance w.r.t cancers?

In the previous subsections “RQ IV)a Which ML classifiers demonstrate reliable performance across all omics modalities?” and “RQ IV)b What DL classifiers demonstrate reliable performance across all omics modalities?”, we comprehensively discussed the performance trends of 15 ML and 20 DL classifiers across 8 distinct omics modalities based on independent testing. However, it is also important to analyze classifiers’ performance across different cancers to understand their generalizability and reliability in cancer molecular subtype classification. Since cancer subtypes exhibit distinct molecular characteristics, some classifiers may excel in specific cancers but struggle in others due to data complexity, sample heterogeneity, or imbalanced subtype distributions. To illustrate this, we investigate RQ V by selecting the highest predictive performance achieved by each classifier for each cancer, regardless of the omics modality based on independent testing. This analysis first identifies the top-performing ML and DL classifiers for each cancer type, followed by an evaluation of the most consistent classifiers that demonstrate robust performance across all cancers.

Figure 5a presents the highest MACC achieved by 15 ML classifiers across 20 cancers. SVM achieves the highest mean MACC of 0.78 and outperforms all other classifiers in 9 cancers: BLCA, BRCA, COAD, ESCA, KIRC, LIHC, SKCM, STAD, and UCEC. LR ranks second with a mean MACC of 0.77 and records the highest performance in 4 cancers: BLCA, ESCA, KIRP, and UCEC. XGB follows closely with a mean MACC of 0.75 and performs best in 3 cancers: GBM, LAML, and ESCA. Other boosting-based classifiers such as HGB and CB also achieve competitive results in some cancers i.e., PRAD, THCA, and ACC. These findings suggest that ensemble, SVM, LR, XGB, and HGB classifiers remain strong contenders for cancer molecular subtype classification across a diverse array of cancers.

Figure 5b presents the highest MACC achieved by 20 DL classifiers across 20 distinct cancers. NN achieves the highest mean MACC of 0.74, with the highest MACC in BLCA, GBM, LAML, and SKCM. MLP follows closely with an mean MACC of 0.72, having the highest MACC across 4 cancers namely, ESCA, LGG, PCPG, and PRAD. DeepGene transformer and CNN follow closely with a mean MACC of 0.71, with DeepGene transformer excelling in PCPG and KIRP, while CNN ranks the highest in ACC, BLCA, LUSC, and PCPG. Among ResNet architectures, ResNet18 achieves the best performance with a mean MACC of 0.70, excelling in BLCA, BRCA, and LUSC. GRU and LSTM achieve mean MACCs of 0.67 and 0.66, respectively, both performing best in BLCA and SKCM. DenseNet architectures (DenseNet264, DenseNet201, DenseNet169, DenseNet161, and DenseNet121) show moderate performance, with mean MACCs between 0.49 and 0.52, without dominating any particular cancer type. Hybrid models such as CNN-RNN, CNN-LSTM, and CNN-GRU perform moderately, with CNN-RNN reaching a mean MACC of 0.62, ranking highest for KIRP.

### RQ VI) Among top-performing classifiers in specific cancers, how do ML and DL methods compare in terms of consistency and suitability for cancer molecular subtype classification?

In the previous subsections, we provided insights into 17 different dataset configurations, where 5 configurations are identified as the most effective across 5 out of the 8 tested omics modalities. Then, in subsections “RQ II & III) How consistently do different omics modalities perform in cancer subtype classificationacross diverse cancers?”–“RQ V) Which specific ML and DL classifiers provide consistent predictive performance w.r.t cancers?”, we evaluated 35 different ML and DL classifiers in terms of 20 distinct cancers and 8 different omics modalities, where 5 ML classifiers (SVM, LR, XGB, HGB, and RF) and 5 DL classifiers (NN, MLP, CNN, ResNet18, ResNet34, RNN, and DEEPGENE) are identified as the most reliable classifiers for cancer molecular subtype classification. Hereby, we address RQ VI by summarizing the top-performing combinations of ML and DL classifiers, dataset configurations, and omics modalities which provide a comprehensive foundation for optimal AI-driven cancer molecular subtype classification.

Table 5 shows the top-performing ML/DL classifiers across 20 cancers where DL classifiers outperform ML classifiers in 11 out of 20 cancers i.e., BLCA, ESCA, KIRP, KIRC, LGG, LUAD, LUSC, PCPG, SKCM, STAD, and UCEC. In contrast, ML models excel in 9 cancers, namely ACC, BRCA, COAD, GBM, HNSC, LAML, LIHC, PRAD, and TCHA. The performance differences between ML and DL classifiers can be attributed to key dataset characteristics such as the number of features, the number of samples, and the number of classes. These key attributes of the benchmark datasets are previously discussed in section 2.2.

**Fig. 6:**
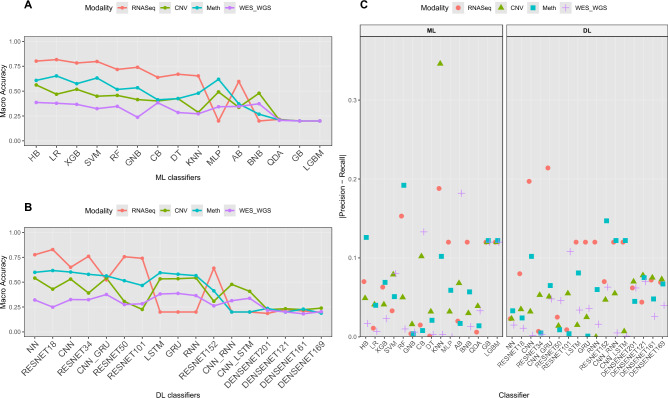
External validation of ML and DL classifiers on the Metabric cohort. (**A**) Macro accuracy of ML classifiers across four omics modalities (RNASeq, CNV, Methylation, WES/WGS). (**B**) Macro accuracy of DL classifiers across the same modalities. (**C**) Precision–Recall disparity (|PR–RE|) for all classifiers, stratified by ML and DL groups.

ML models such as SVM, HGB, XGB, and RF tend to excel when datasets have fewer features, samples, and class labels. A lower feature count, typically between 131 and 10,000, reduces the need for deep feature extraction, making traditional ML models a more efficient choice. For instance, in BRCA (miRNA-GA-gene, SVM, MACC = 0.88) and UCEC (HumanMethylation27, LSTM, MACC = 0.88), where the feature count is relatively low (600–15,000), SVM performs well by effectively separating classes with kernel methods without requiring complex hierarchical feature learning. ML models also outperform DL models when the datasets have a limited number of samples as DL classifiers require larger datasets to avoid overfitting. For instance, HNSC (RPPA, GNB, MACC = 0.71), and LIHC (miRNA, SVM, MACC = 0.86) where the total number of samples are 100-200, ML classifiers provides robust performance by effectively handling small-sample datasets (Fig. 6).

DL models like ResNet18, ResNet34, ResNet101, CNN, RNN, and DEEPGENE excel with high-dimensional datasets (>20,000 features), larger sample sizes ($\ge$ 150), and multi-class classification. They leverage hierarchical feature representations that traditional ML classifiers struggle to model. For example, in KIRP (CNV, DEEPGENE, MACC = 0.887) with 24,776 features and 161 samples, DL outperforms ML by capturing complex patterns despite the moderate sample size. Similarly, in LUAD (HumanMethylation27, ResNet101, MACC = 1.00) with 24,979 features and 226 samples, DL effectively handles high-dimensional data. LUSC (HT-HG-U133A, ResNet34, MACC = 0.92) further demonstrates DL’s superiority in cancer molecular subtype classification (12,042 features, 104 samples). DL models generalize well with>150 samples where they prevent overfitting and capture intricate feature relationships. For instance, in PCPG (HiSeqV2-percentile, MLP, MACC = 1.00) with 20,501 features and 173 samples, and STAD (somatic mutation, DeepGene, MACC = 0.83) with 40,543 features and 383 samples, DL outperforms ML by modeling complex feature interactions across class distributions. Additionally, DL models perform well in datasets with six or more classes, effectively distinguishing cancer molecular subtypes. In LGG (7 classes, 513 samples), DL excels in multi-class classification, while in GBM (7 classes, 577 samples), ML (XGB) is an exception where sample size and feature dimensionality likely outweigh class complexity.

### RQ VIII) Do the conclusions drawn in RQ I, II, and III remain consistent when evaluated on an independent external validation dataset?

This section evaluates whether the key findings from RQ I–III generalize beyond the internal TCGA datasets. Particularly, first it presents the performance trends of ML and DL classifiers and then it also highlights PR-RE disparity of the classifiers in terms of the external datasets.

TCGA^68^ provides a diverse and modality-complete set of omics datasets, external cohorts rarely offer uniform coverage of all modalities, configurations, and subtype annotations. Using heterogeneous or partially matched datasets could introduce substantial biases due to differences in preprocessing pipelines, normalization methods, and sample labeling schemes. Moreover, addressing RQ I would require consistent availability of all omics modalities and their configurations across identical cancer cohorts, which is currently highly impractical outside TCGA. Therefore, to ensure a fair and modality-consistent comparison, we focus our external validation efforts on RQ II and RQ III using the Metabric Breast cancer cohort^72^.

Figures 6A–B present the MACC trends of ML and DL classifiers across all four modalities. Classifiers that perform strongly on TCGA, such as SVM, LR, HGB, NN, CNN, and ResNet18, continue to demonstrate competitive MACC on Metabric cohort. Classifiers trained on RNASeq, and Meth. again show superior predictive performance, which reflects the modality-wise stability observed in RQ II. These results confirm that RNASeq and Meth–based datasets preserve discriminative molecular signals even under platform and cohort shifts.

Figure 6C illustrates the PR–RE disparity across classifiers and modalities. Consistent with RQ III, modalities with lower MACC particularly WES_WGS exhibit higher PR–RE imbalance, indicating persistent susceptibility to prediction bias. Conversely, RNASeq and CNV maintain low disparity values along with high overall predictive performance, supporting their robustness and balanced subtype classification. Classifiers previously identified as stable (e.g., SVM, LR, NN, CNN) continue to produce low PR–RE disparity.

Overall, the external validation results confirm that the key conclusions from RQ II and RQ III remain consistent in an independent clinical dataset: (i) the relative performance ranking of modalities persists, with RNASeq and Meth. being the most informative and WES/WGS the least stable, and (ii) modality-specific biases observed in TCGA reappear in Metabric. These findings demonstrate that the benchmarking insights generalize beyond TCGA and validate the robustness and reproducibility of the proposed framework across heterogeneous real-world datasets.

## Discussion

This study presents a comprehensive benchmarking framework for evaluating the predictive performance of 35 different ML/DL classifiers across 153 datasets spanning 8 omics modalities and 20 cancer types for cancer molecular subtype classification. The results provide critical insights into the effectiveness of different omics modalities, data configurations, and AI classifiers for cancer molecular subtype classification. In this context, we discuss the key findings, their implications, and future directions for research in this domain.

The first critical step in designing effective and accurate predictive pipelines for cancer molecular subtype classification is the selection of optimal omics modalities and their configurations. Among data configurations, Gistic2-all-data-by-genes (CNV) and HiSeqV2 (RNASeq) consistently outperform others, emphasizing the value of granular, continuous genomic representations. HiSeqV2, HiSeqV2-PANCAN, and HiSeqV2-percentile show nearly identical performance, confirming RNASeq’s robustness. In RPPA, unprocessed data outperforms RPPA-RBN by preserving essential signals. The array modality varies by configuration, while in Meth., HM27 surpasses HM450 due to a higher signal-to-noise ratio and lower overfitting risk. These findings highlight the impact of dataset configurations on classifier performance in cancer molecular subtype classification.

Our large-scale and comprehensive showed that miRNA, Meth., Exon, CNV, and RNASeq (gene expression), are the most informative modalities with the highest MACC in 14 cancers namely, BLCA, BRCA, LAML, LIHC, ESCA, KIRP, LGG, SKCM, COAD, THCA, PCPG, and PRAD. However, SNP and RPPA modalities showed lower predictive performance and higher PR-RE disparities which indicates challenges in leveraging these modalities for accurate cancer molecular subtype classification.

In terms of classifiers, large scale performance analyses identified SVM, LR, and XGB as the most consistent ML classifiers across multiple cancers, while ResNet18, CNN, and DEEPGENE emerged as the top-performing DL models. These classifiers demonstrated strong generalizability, achieving high MACC across diverse datasets and modalities. However, their performance varied depending on the cancer type and dataset characteristics, underscoring the need for modality-specific model selection. A comparison of ML and DL classifiers showed that ML models like SVM, LR, and XGB perform well in datasets with moderate feature counts and small sample sizes, effectively handling class imbalance and avoiding overfitting. In contrast, DL models such as ResNet, CNN, and DEEPGENE excel in high-dimensional datasets with larger sample sizes by capturing complex patterns through hierarchical feature representations. This distinction highlights the strengths of ML models for low-dimensional data and DL models for large-scale, multi-class classification tasks in omics research.

While DL models such as CNN, ResNet, and DEEPGENE have shown strong performance, Transformer-based models are emerging as a potential alternative for cancer molecular subtype classification i.e., DEEPGENE. Transformers have demonstrated state-of-the-art results in various domains but face significant challenges in omics applications. For instance, DEEPGENE relies on CNNs to transform features before self-attention is applied, which may lead to the loss of critical gene associations and other information. In addition, DEEPGENE is trained using supervised learning, requiring large labeled datasets. However, cancer subtype data is often limited, particularly for rare subtypes. Moreover, one major drawback of Transformers is their inability to handle extremely high-dimensional input directly. Standard architectures impose a maximum input length constraint (e.g., 512 tokens), which is insufficient for omics datasets containing tens of thousands of features (e.g., gene expression, methylation, CNV data). On the basis of such challenges, future work should investigate unsupervised or self-supervised pretraining approaches to reduce dependency on large labeled datasets, and effective embedding based strategies such that discriminatory and useful information is retained in the final representation of the data.

In terms of cancer molecular subtype classification, two different challenges are observed i.e., class imbalance, and modality bias. Class imbalance remains a significant challenge in cancer molecular subtype classification. For example, in BRCA, the Luminal A subtype (566 samples) is overrepresented compared to the Normal subtype (40 samples). This imbalance can lead to biased predictions, as classifiers may prioritize majority classes. Techniques such as stratified sampling and data augmentation should be explored to mitigate this issue. Modality-specific biases were also observed, particularly in SNP and RPPA datasets, where classifiers exhibited high PR-RE disparities. These biases may stem from noise, technical variability, or insufficient representation of certain subtypes in the data. Future work should focus on developing robust preprocessing pipelines and normalization techniques to address these challenges.

While this study provides valuable insights, several limitations should be acknowledged. First, the datasets used in this benchmark are primarily derived from TCGA and Metabric, which may not fully capture the heterogeneity of cancer subtypes across different populations. Future studies should incorporate datasets from diverse sources, such as GEO and cBioPortal, to enhance generalizability. Second, the study focused on single-omics modalities, whereas multi-omics integration could provide a more comprehensive understanding of cancer molecular subtypes. Developing AI models capable of integrating multiple omics layers (e.g., combining CNV, methylation, and RNAseq) is a promising direction for future research.

Additionally, the study did not explore the impact of hyperparameter tuning on classifier performance. Optimizing hyperparameters for specific modalities and cancers could further improve predictive accuracy. Finally, the interpretability of AI models remains a critical challenge. While DL models often outperform ML classifiers, their “black-box” nature limits their adoption in clinical settings. Future work should focus on developing explainable AI (XAI) techniques to enhance the transparency and trustworthiness of these models.

## Conclusion

This study in hand presents a comprehensive benchmarking analysis of AI-driven cancer molecular subtype classification across 20 different cancers using 8 distinct omics modalities and 35 ML/DL classifiers. Our findings highlight that certain omics modalities, such as RNASeq, miRNA, Exon, and CNV, provide more consistent and reliable performance in cancer molecular subtype classification, while others, such as SNP, RPPA, and Array, exhibit greater variability and require further optimization. Among classifiers, ML models like SVM, XGB, and HGB tend to perform well on smaller datasets with lower feature counts, whereas DL models such as ResNet18, CNN-RNN, and CNN-GRU demonstrate superior performance on high-dimensional datasets with large sample sizes. Additionally, the study underscores the disparity between precision and recall as a key challenge in classification tasks, particularly for underperforming modalities. Overall, our benchmarking results provide critical insights into the strengths and limitations of different omics modalities and AI classifiers in cancer molecular subtype classification. By identifying the most effective classifier-modality combinations, this study serves as a foundation for developing robust and standardized AI pipelines for precision oncology. Future work should focus on mitigating modality-specific biases, improving performance on challenging data types, integrating multi-omics approaches to enhance classification accuracy and generalizability, and the application of transformer-based classifiers for cancer molecular subtype classification.

## Supplementary Information

Supplementary Information 1.

Supplementary Information 2.

## Data Availability

The dataset(s) supporting the results of this article are available through UCSC Xena https://xenabrowser.net/datapages/.
