# Supplementary material for: Multi-omics driven computational framework for cancer molecular subtype classification
Source: Sci Rep. 2025 Dec 19;15:44141. doi: 10.1038/s41598-025-32051-5 (PMC12717050; doi:10.1038/s41598-025-32051-5)
Supplement: Supplementary file 1 — Supplementary Information 1. [file 41598_2025_32051_MOESM1_ESM.pdf]

Supplementary Information

1. How are the datasets of DNA methylation preprocessed?

Large datasets like HumanMethylation450 (HM450) and HumanMethylation27 (HM27) contain high-dimensional methylation data, measuring DNA methylation at 485,000 and 27,000 CpG sites, respectively. This results in datasets with an extremely high number of features, which can be computationally challenging to analyze directly. To overcome this, the TCGA-Assembler2 package in R (Wei et al., 2018) provides a streamlined preprocessing pipeline that reduces dimensionality while retaining biologically relevant information. The pipeline assumes that raw beta values have been preprocessed and normalized using external tools, such as SWAN (Subset-quantile Within Array Normalization) or BMIQ (Beta Mixture Quantile Normalization), which are specifically designed for Illumina methylation arrays. These normalization methods correct technical variations, such as batch effects and probe-specific biases, ensuring that the data are comparable across samples and platforms.

A critical step in reducing the high dimensionality of HM450 and HM27 data is the aggregation of methylation values at the gene level. TCGA-Assembler2 uses the CalculateSingleValueMethylationData function to summarize methylation beta values for specific genomic regions, such as promoter regions (e.g., TSS1500 and TSS200). For example, methylation levels within TSS1500 (1500 base pairs upstream of the transcription start site) and TSS200 (200 base pairs upstream) are averaged to produce a single gene-level beta value. This approach reduces the number of features from hundreds of thousands of CpG sites to a more manageable number of genes, while preserving the biological significance of methylation patterns in regulatory regions. By focusing on promoter regions, which are critical for gene regulation, this step ensures that the summarized data are both interpretable and relevant for downstream analysis.

Finally, TCGA-Assembler2 addresses the challenge of batch effects and technical variations, which are common in large-scale methylation studies. While the package does not include built-in functions for batch effect correction or quality control (QC), it assumes that users will perform these steps externally using tools like ComBat (for batch effect correction) or the minfi package (for QC). The output of this preprocessing pipeline is a gene-level methylation matrix, where rows represent genes and columns represent samples. This matrix is significantly smaller in size compared to the original dataset, making it computationally efficient for downstream tasks such as identifying differentially methylated genes (DMGs), clustering samples, or integrating methylation data with other omics data. By reducing the dimensionality and improving data quality, TCGA-Assembler2 enables researchers to extract meaningful insights from HM450 and HM27 datasets without being overwhelmed by the high number of features.

[1] Wei, L., Jin, Z., Yang, S., Xu, Y., Zhu, Y., & Ji, Y. (2018). TCGA-assembler 2: software pipeline for retrieval and processing of TCGA/CPTAC data. Bioinformatics (Oxford, England), 34(9), 1615–1617. https://doi.org/10.1093/bioinformatics/btx812

2. What are the standard preprocessing methods used?

   a. Samples without valid subtype labels were removed.
   b. Samples containing >80% zero or missing values were excluded to avoid sparsity-driven biases.
   c. Classes with fewer than 10 samples were removed to prevent unreliable model training.

Supplementary Table S1: A comprehensive collection of 153 benchmark datasets for cancer molecular subtype classification that encompasses 20 distinct cancers and spans 17 unique data configurations across 8 omics modalities.

Cancer	Modality	Configuration	Classes	Total Samples	Samples Per Class	No of Features

PRAD	miRNA	miRNA HiSeq gene	8	330	ERG: 151, SPOP: 37, 4 FLI1: 4, Other: 85, FOXA1: 9, ETV1: 27, ETV4: 14, IDH1: 3	633
	RNASeq	HiSeqV2	8	333	ETV1: 28, ERG: 152, Other: 86, IDH1: 3, ETV4: 14, SPOP: 37, FOXA1: 9, 4 FLI1: 4	20530
		HiSeqV2 PANCAN	8	333	ETV1: 28, ERG: 152, Other: 86, IDH1: 3, ETV4: 14, SPOP: 37, FOXA1: 9, 4 FLI1: 4	20530
		HiSeqV2 percentile	8	333	ETV1: 28, ERG: 152, Other: 86, IDH1: 3, ETV4: 14, SPOP: 37, FOXA1: 9, 4 FLI1: 4	20501
	Exon	HiSeqV2 exon	8	333	ETV1: 28, ERG: 152, Other: 86, IDH1: 3, ETV4: 14, SPOP: 37, FOXA1: 9, 4 FLI1: 4	239322
	RPPA	RPPA	8	250	SPOP: 23, ERG: 116, IDH1: 3, ETV1: 23, Other: 65, FOXA1: 7, ETV4: 10, 4 FLI1: 3	218
	SNP/ INDEL	mc3 gene level	8	331	ERG: 151, Other: 86, ETV1: 28, FOXA1: 9, 4 FLI1: 3, ETV4: 14, IDH1: 3, SPOP: 37	40543
	CNV	Gistic2 all data by genes	8	333	ERG: 152, Other: 86, ETV1: 28, FOXA1: 9, 4 FLI1: 4, ETV4: 14, IDH1: 3, SPOP: 37	24776
		Gistic2 all thresholded	8	333	ERG: 152, Other: 86, ETV1: 28, FOXA1: 9, 4 FLI1: 4, ETV4: 14, IDH1: 3, SPOP: 37	24776
	RPPA	RPPA	5	311	CIN: 178, GS: 39, MSI: 62, EBV: 26, HM SNV: 6	218
	SNP/ INDEL	mc3 gene level	5	383	CIN: 223, GS: 50, MSI: 73, EBV: 30, HM SNV: 7	40543

STAD	CNV	Gistic2 all data by genes	5	383	CIN: 223, GS: 50, MSI: 73, EBV: 30, HM SNV: 7	24776
		Gistic2 all thresholded	5	383	CIN: 223, GS: 50, MSI: 73, EBV: 30, HM SNV: 7	24776
	Methylation	Human Methylation450	5	343	'GI.CIN': 202, 'GI.EBV': 59, 'GI.GS': 29, 'GI.MSI': 46, HM SNV:7	20789
PCPG	miRNA	miRNA HiSeq gene	4	173	Kinase signaling: 68, Wnt altered: 22, Pseudohypoxia: 61, Cortical admixture: 22	796
	RNASeq	HiSeqV2	4	173	Kinase signaling: 68, Pseudohypoxia: 61, Cortical admixture: 22, Wnt altered: 22	20530
		HiSeqV2 PANCAN	4	173	Kinase signaling: 68, Pseudohypoxia: 61, Cortical admixture: 22, Wnt altered: 22	20530
		HiSeqV2 percentile	4	173	Kinase signaling: 68, Pseudohypoxia: 61, Cortical admixture: 22, Wnt altered: 22	20501
	Exon	HiSeqV2 exon	4	173	Kinase signaling: 68, Pseudohypoxia: 61, Cortical admixture: 22, Wnt altered: 22	239322
	RPPA	RPPA	4	76	Pseudohypoxia: 25, Kinase signaling: 34, Wnt altered: 10, Cortical admixture: 7	219
	SNP/ INDEL	PCPG mc3 gene level	4	173	Kinase signaling: 68, Wnt altered: 22, Pseudohypoxia: 61, Cortical admixture: 22	40543
	CNV	Gistic2 all data by genes	4	159	Kinase signaling: 62, Wnt altered: 21, Pseudohypoxia: 54, Cortical admixture: 22	24776

		Gistic2 all thresholded	4	159	Kinase signaling: 62, Wnt altered: 21, Pseudohypoxia: 54, Cortical admixture: 22	24776
HNSC	RPPA	RPPA	4	200	Mesenchymal: 58, Classical: 40, Basal: 66, Atypical: 36	131
	RPPA	RPPA	4	200	Basal: 66, Mesenchymal: 58, Atypical: 36, Classical: 40	238
	SNP/ INDEL	mc3 gene level	4	277	Mesenchymal: 74, Basal: 87, Atypical: 67, Classical: 49	40543
	CNV	Gistic2 all thresholded	4	279	Mesenchymal: 75, Basal: 87, Atypical: 68, Classical: 49	24776
		Gistic2 all data by genes	4	279	Mesenchymal: 75, Basal: 87, Atypical: 68, Classical: 49	24776
	Methylation	Human Methylation450	4	279	Mesenchymal: 75, Basal: 49, Atypical: 87, Classical: 68	20789
BLCA	miRNA	miRNA HiSeq gene	4	129	BLCA.2: 42, BLCA.1: 41, BLCA.3: 31, BLCA.4: 15	810
	RNASeq	HiSeqV2	4	129	BLCA.2: 42, BLCA.4: 15, BLCA.3: 31, BLCA.1: 41	20530
		HiSeqV2 PANCAN	4	129	BLCA.2: 42, BLCA.4: 15, BLCA.3: 31, BLCA.1: 41	20530
		HiSeqV2 percentile	4	129	BLCA.2: 42, BLCA.4: 15, BLCA.3: 31, BLCA.1: 41	20501

	Exon	HiSeqV2 exon	4	129	BLCA.2: 42, BLCA.1: 41, BLCA.3: 31, BLCA.4: 15	78722
	Methylation	Human Methylation450	4	129	BLCA.2: 42, BLCA.1: 41, BLCA.3: 31, BLCA.4: 15	20789
	RPPA	RPPA	4	118	BLCA.1: 36, BLCA.4: 15, BLCA.2: 38, BLCA.3: 29	131
		RPPA	4	118	BLCA.2: 38, BLCA.1: 36, BLCA.3: 29, BLCA.4: 15	222
	SNP/ INDEL	mc3 gene level	4	129	BLCA.1: 41, BLCA.4: 15, BLCA.2: 42, BLCA.3: 31	40543
	CNV	Gistic2 all thresholded	4	126	BLCA.1: 41, BLCA.4: 15, BLCA.2: 41, BLCA.3: 29	24776
		Gistic2 all data by genes	4	126	BLCA.1: 41, BLCA.4: 15, BLCA.2: 41, BLCA.3: 29	24776
SKCM	CNV	Gistic2 all thresholded	5	266	RAS Hotspot Mutants: 81, Triple WT: 38, BRAF Hotspot Mutants: 118, : 6, NF1 Any Mutants: 23	24776
	Methylation	Human Methylation450	5	333	RAS Hotspot Mutants: 92, Triple WT: 46, BRAF Hotspot Mutants: 150, : 17, NF1 Any Mutants: 28	20789
COAD	miRNA	miRNA GA gene	4	100	HM SNV: 4, GS: 17, CIN: 62, MSI: 17	617
	RNASeq	GAV2	4	95	CIN: 59, MSI: 16, GS: 16, HM SNV: 4	20530

	Exon	GAV2 exon	4	95	CIN: 59, MSI: 16, GS: 16, HM SNV: 4	239322
	RPPA	RPPA	4	245	CIN: 152, MSI: 45, GS: 43, HM SNV: 5	131
	SNP/ INDEL	mc3 gene level	4	268	CIN: 178, MSI: 50, GS: 34, HM SNV: 6	40543
	CNV	Gistic2 all thresholded	4	341	CIN: 226, MSI: 60, GS: 49, HM SNV: 6	24776
		Gistic2 all data by genes	4	341	CIN: 226, MSI: 60, GS: 49, HM SNV: 6	24776
LAML	miRNA	miRNA GA gene	8	187	NA: 9, 7: 20, 4: 37, 1: 14, 2: 21, 6: 35, 3: 18, 5: 33	544
	RNASeq	HiSeqV2 PANCAN	7	172	3: 18, 4: 35, 1: 14, 5: 32, 6: 35, 2: 20, 7: 18	20530
		GA	7	178	6: 35, 5: 33, 1: 14, 2: 21, 7: 20, 4: 37, 3: 18	20113
		HiSeqV2 percentile	7	172	3: 18, 4: 35, 1: 14, 5: 32, 6: 35, 2: 20, 7: 18	20501
		HiSeqV2	7	172	3: 18, 4: 35, 1: 14, 5: 32, 6: 35, 2: 20, 7: 18	20530
	Exon	GA exon	7	178	3: 18, 4: 37, 1: 14, 5: 33, 6: 35, 2: 20, 7: 20	219296

		HiSeqV2 exon	7	172	3: 18, 4: 35, 1: 14, 5: 32, 6: 35, 2: 20, 7: 18	155775
	SNP/ INDEL	mutation wustl gene	8	184	NA: 9, 3: 18, 5: 32, 4: 37, 6: 34, 1: 13, 2: 21, 7: 20	43785
	CNV	Gistic2 all data by genes	8	178	3: 18, 5: 31, 4: 36, 6: 33, 1: 13, 2: 19, 7: 20, NA: 8	24776
		Gistic2 all thresholded	8	178	3: 18, 5: 31, 4: 36, 6: 33, 1: 13, 2: 19, 7: 20, NA: 8	24776
LGG	SNP/ INDEL	LGG mc3 gene level	6	511	G high: 234, Mesenchymal like: 45, Codel: 173, Classic like: 23, G low: 12, PA like: 24	40543
	CNV	Gistic2 all thresholded	7	513	G high: 233, Mesenchymal like: 45, Codel: 172, Classic like: 23, G low: 12, PA like: 25, NA: 3	24776
		Gistic2 all data by genes	7	513	G high: 233, Mesenchymal like: 45, Codel: 172, Classic like: 23, G low: 12, PA like: 25, NA: 3	24776
READ	miRNA	miRNA HiSeq gene	4	84	CIN: 74, MSI: 2, GS: 6, HM SNV: 2	773
	RPPA	RPPA	4	89	CIN: 77, GS: 7, MSI: 3, HM SNV: 2	222
	RPPA	RPPA	4	88	CIN: 76, GS: 7, HM SNV: 2, MSI: 3	131
	SNP/ INDEL	mc3 gene level	4	84	CIN: 74, GS: 6, MSI: 2, HM SNV: 2	40543

	CNV	Gistic2 all data by genes	4	118	CIN: 102, GS: 9, HM SNV: 4, MSI: 3	24776
		Gistic2 all thresholded	4	118	CIN: 102, GS: 9, HM SNV: 4, MSI: 3	24776
UCEC	miRNA	miRNA GA gene	5	126	CN LOW: 53, MSI: 32, NA: 6, POLE: 16, CN HIGH: 19	618
	RPPA	RPPA	5	431	CN HIGH: 126, MSI: 95, POLE: 68, CN LOW: 116, NA: 26	222
		RPPA	5	397	POLE: 61, NA: 26, CN HIGH: 110, MSI: 89, CN LOW: 111	131
	SNP/ INDEL	mc3 gene level	5	444	CN HIGH: 143, CN LOW: 115, POLE: 69, NA: 15, MSI: 102	40543
	CNV	Gistic2 all data by genes	5	530	CN HIGH: 160, CN LOW: 144, POLE: 79, MSI: 124, NA: 23	24776
		Gistic2 all thresholded	5	530	CN HIGH: 160, CN LOW: 144, POLE: 79, MSI: 124, NA: 23	24776
	Methylation	Human Methylation27	5	117	CN HIGH: 21, CN LOW: 42, POLE: 15, MSI: 32, NA: 7	12778
	Methylation	Human Methylation450	5	423	CN HIGH: 140, CN LOW: 102, POLE: 64, MSI: 93, NA: 24	20789
ACC	miRNA	miRNA HiSeq gene	3	79	intermediate: 27, high: 20, low: 32	774

	RNASeq	HiSeqV2 PANCAN	3	78	intermediate: 27, high: 19, low: 32	20530
		HiSeqV2	3	78	intermediate: 27, high: 19, low: 32	20530
		HiSeqV2 percentile	3	78	intermediate: 27, high: 19, low: 32	20501
	Methylation	Human Methylation450	3	79	intermediate: 27, low: 32, high: 20	20789
	SNP/ INDEL	ACC mc3 gene level	4	91	high: 20, low: 32, intermediate: 27, NA: 12	40543
	CNV	Gistic2 all data by genes	4	89	high: 20, low: 32, intermediate: 25, NA: 12	24776
		Gistic2 all thresholded	4	78	high: 18, low: 28, intermediate: 22, NA: 10	24776
LUAD	RPPA	RPPA	6	181	2: 28, 5: 42, 1: 19, 6: 32, 4: 22, 3: 38	238
		RPPA	6	181	5: 42, 1: 19, 4: 22, 3: 38, 2: 28, 6: 32	131
	SNP/ INDEL	mc3 gene level	6	226	5: 51, 6: 41, 1: 22, 2: 31, 3: 49, 4: 32	40543
	CNV	Gistic2 all data by genes	6	230	5: 52, 6: 41, 1: 22, 2: 32, 3: 51, 4: 32	24776

		Gistic2 all thresholded	6	230	5: 52, 6: 41, 1: 22, 2: 32, 3: 51, 4: 32	24776
	Methylation	Human Methylation450	6	188	5: 44, 6: 35, 1: 18, 2: 22, 3: 42, 4: 27	20789
LIHC	miRNA	miRNA HiSeq gene	4	191	iCluster:2: 55, iCluster:3: 63, iCluster:1: 65, NA: 8	747
	RNASeq	HiSeqV2	4	193	iCluster:2: 55, iCluster:3: 63, iCluster:1: 65, NA: 10	20530
		HiSeqV2 percentile	4	193	iCluster:2: 55, iCluster:3: 63, iCluster:1: 65, NA: 10	20501
		HiSeqV2 PANCAN	4	193	iCluster:2: 55, iCluster:3: 63, iCluster:1: 65, NA: 10	20530
	Exon	HiSeqV2 exon	4	193	iCluster:2: 55, iCluster:3: 63, iCluster:1: 65, NA: 10	239322
	Methylation	Human Methylation450	4	196	iCluster:2: 55, iCluster:3: 63, iCluster:1: 65, NA: 13	20789
	RPPA	RPPA	4	162	iCluster:1: 56, iCluster:3: 48, iCluster:2: 46, NA: 12	219
	SNP/ INDEL	mc3 gene level	4	182	iCluster:3: 58, iCluster:1: 60, iCluster:2: 52, NA: 12	40543
	CNV	Gistic2 all thresholded	4	191	iCluster:1: 65, iCluster:3: 63, iCluster:2: 55, NA: 8	24776

		Gistic2 all data by genes	4	191	iCluster:1: 65, iCluster:3: 63, iCluster:2: 55, NA: 8	24776
THCA	miRNA	miRNA HiSeq gene	6	493	1: 133, 5: 91, NA: 14, 2: 67, 4: 106, 3: 82	763
	RNASeq	HiSeqV2 percentile	6	494	2: 67, 5: 93, 4: 106, 1: 134, 3: 82, NA: 12	20501
		HiSeqV2 PANCAN	6	494	2: 67, 5: 93, 4: 106, 1: 134, 3: 82, NA: 12	20530
		HiSeqV2	6	494	2: 67, 5: 93, 4: 106, 1: 134, 3: 82, NA: 12	20530
	Exon	HiSeqV2 exon	6	494	2: 67, 5: 93, 4: 106, 1: 134, 3: 82, NA: 12	239322
	Methylation	Human Methylation450	6	496	1: 134, 4: 106, 5: 93, 3: 82, 2: 67, NA: 14	20789
	RPPA	RPPA	6	368	1: 108, 4: 75, 5: 72, 2: 45, 3: 57, NA: 11	260
	SNP/ INDEL	mc3 gene level	6	481	1: 127, 2: 66, 4: 103, 3: 79, 5: 93, NA: 13	40543
	CNV	Gistic2 all data by genes	6	492	1: 132, 2: 67, 3: 82, 4: 105, NA: 14, 5: 92	24776
		Gistic2 all thresholded	6	492	1: 132, 2: 67, 3: 82, 4: 105, NA: 14, 5: 92	24776

GBM	Array	AgilentG4502A 072	6	473	Mesenchymal like: 135, NA: 194, G low: 8, LGm6 GBM: 26, Classic like: 99, G high: 11	17814
		HT HG U133A	7	529	Classic like: 99, LGm6 GBM: 37, Mesenchymal like: 144, NA: 223, G low: 13, G high: 12, Codel: 1	12042
		AgilentG4502A 071	7	101	Classic like: 15, NA: 41, LGm6 GBM: 12, G low: 5, Mesenchymal like: 25, G high: 2, Codel: 1	17814
	Methylation	Human Methylation450	5	141	LGm6 GBM: 13, NA: 13, Mesenchymal like: 56, Classic like: 53, G high: 6	20789
	RPPA	RPPA	6	215	Mesenchymal like: 71, NA: 50, LGm6 GBM: 14, G low: 5, Classic like: 71, G high: 4	131
	SNP/ INDEL	mc3 gene level	6	314	Mesenchymal like: 117, LGm6 GBM: 22, G low: 6, Classic like: 99, NA: 62, G high: 8	40543
	CNV	Gistic2 all thresholded	7	577	Mesenchymal like: 164, Classic like: 120, G low: 13, LGm6 GBM: 40, NA: 224, Codel: 1, G high: 15	24776
		Gistic2 all data by genes	7	577	Mesenchymal like: 164, Classic like: 120, G low: 13, LGm6 GBM: 40, NA: 224, Codel: 1, G high: 15	24776
ESCA	RPPA	RPPA	5	116	ESCC: 72, CIN: 40, MSI: 1, GS: 1, HM SNV: 2	219
	SNP/ INDEL	mc3 gene level	5	169	CIN: 74, HM SNV: 2, ESCC: 90, GS: 1, MSI: 2	40543

	CNV	Gistic2 all thresholded	5	169	CIN: 74, HM SNV: 2, ESCC: 90, GS: 1, MSI: 2	24776
		Gistic2 all data by genes	5	169	CIN: 74, HM SNV: 2, ESCC: 90, GS: 1, MSI: 2	24776
KIRC	miRNA	miRNA GA gene	5	233	1: 69, 2: 52, 3: 49, 4: 52, NA: 11	581
	RPPA	RPPA	5	408	3: 89, 1: 137, 2: 87, NA: 23, 4: 72	131
		RPPA	5	408	4: 72, 1: 137, 2: 87, 3: 89, NA: 23	232
	SNP/ INDEL	mc3 gene level	5	294	3: 59, 1: 109, 2: 50, NA: 21, 4: 55	40543
	CNV	Gistic2 all thresholded	5	436	1: 145, 3: 92, 2: 90, NA: 23, 4: 86	24776
		Gistic2 all data by genes	5	436	1: 145, 3: 92, 2: 90, NA: 23, 4: 86	24776
	Methylation	Human Methylation450	5	253	1: 91, 3: 53, 2: 36, NA: 19, 4: 54	20789
KIRP	RPPA	RPPA	4	125	C1: 71, C2a: 27, C2c CIMP: 9, C2b: 18	220
	SNP/ INDEL	mc3 gene level	4	158	C2a: 34, C1: 93, C2c CIMP: 9, C2b: 22	40543

	CNV	Gistic2 all data by genes	4	161	C2a: 35, C1: 95, C2c CIMP: 9, C2b: 22	24776
	CNV	Gistic2 all thresholded	4	161	C2a: 35, C1: 95, C2c CIMP: 9, C2b: 22	24776
BRCA	miRNA	miRNA HiSeq gene	5	747	LumA: 390, Basal: 131, Her2: 58, LumB: 136, Normal: 32	707
		miRNA GA gene	5	319	LumA: 167, LumB: 70, Basal: 51, Her2: 23, Normal: 8	593
	RNASeq	HiSeqV2	5	1097	Basal: 192, Her2: 82, LumA: 566, LumB: 217, Normal: 40	20530
		HiSeqV2 percentile	5	1097	Basal: 192, Her2: 82, LumA: 566, LumB: 217, Normal: 40	20501
		HiSeqV2 PANCAN	5	1097	Basal: 192, Her2: 82, LumA: 566, LumB: 217, Normal: 40	20530
	Exon	HiSeqV2 exon	5	1097	Basal: 192, Her2: 82, LumA: 566, LumB: 217, Normal: 40	239322
	Array	AgilentG4502A 073	5	529	Her2: 46, LumA: 268, Basal: 96, LumB: 108, Normal: 11	17814
	Methylation	Human Methylation27	5	314	LumA: 146, Her2: 36, LumB: 70, Basal: 56, Normal: 6	12778
		Human Methylation450	5	781	LumA: 418, Her2: 46, LumB: 147, Basal: 136, Normal: 34	20789

	RPPA	RPPA	5	742	LumA: 358, Basal: 130, Her2: 67, LumB: 167, Normal: 20	131
		RPPA	5	885	Basal: 157, LumA: 439, LumB: 184, Her2: 75, Normal: 30	222
	SNP/ INDEL	mc3 gene level	5	789	LumA: 413, Her2: 54, LumB: 145, Normal: 32, Basal: 145	40543
	CNV	Gistic2 all data by genes	5	1078	LumA: 556, Her2: 81, LumB: 216, Normal: 39, Basal: 186	24776
		Gistic2 all thresholded	5	1078	LumA: 556, Her2: 81, LumB: 216, Normal: 39, Basal: 186	24776
USC	miRNA	miRNA GA gene	4	107	secretory: 20, classical: 44, primitive: 14, basal: 29	660
	Array	HT HG U133A	4	104	classical: 37, primitive: 16, secretory: 21, basal: 30	12042
		AgilentG4502A 073	4	121	classical: 45, primitive: 19, secretory: 25, basal: 32	17814
	RPPA	RPPA	4	112	classical: 38, basal: 28, secretory: 28, primitive: 18	131
		RPPA	4	112	classical: 38, primitive: 18, basal: 28, secretory: 28	238
	SNP/ INDEL	mc3 gene level	4	173	basal: 41, classical: 63, secretory: 42, primitive: 27	40543

	CNV	Gistic2 all data by genes	4	178	basal: 43, classical: 65, secretory: 43, primitive: 27	24776
		Gistic2 all thresholded	4	178	basal: 43, classical: 65, secretory: 43, primitive: 27	24776

Supplementary Table S2: (Fig. 2.) MACC values of top performing classifiers in terms of 20 different configurations of 8 distinct omics modalities across 20 different cancers.

**Cancer**	**Modality**	**Scale**	**Type**	**Classifier**	**ACC**	**PR**	**RE**	**F1**	**MCC**	**AUROC**
ACC	Meth.	HumanMethylation450	ML	HGB	1	1	1	1	1	1
ACC	CNV	Gistic2-all-data-by-genes	DL	RESNET34	0.87	0.9	0.87	0.87	0.94	0.95
ACC	CNV	Gistic2-all-thresholded	DL	NN	0.69	0.71	0.69	0.7	0.85	0.75
ACC	RNASeq	HiSeqV2	ML	SVM	0.83	0.9	0.83	0.84	0.93	0.95
ACC	RNASeq	HiSeqV2-PANCAN	ML	SVM	0.83	0.9	0.83	0.84	0.93	0.95
ACC	RNASeq	HiSeqV2-percentile	DL	RESNET18	0.8	0.8	0.8	0.8	0.9	0.92
ACC	SNP	mc3-gene-level	ML	CNB	0.53	0.3	0.53	0.37	0.74	0.67
ACC	miRNA	miRNA-HiSeq-gene	ML	BNB	0.8	0.85	0.8	0.82	0.9	0.9
BLCA	Meth.	HumanMethylation450	ML	HGB	0.79	0.79	0.79	0.79	0.93	0.93
BLCA	CNV	Gistic2-all-data-by-genes	DL	NN	0.66	0.71	0.66	0.67	0.87	0.81
BLCA	CNV	Gistic2-all-thresholded	ML	SVM	0.57	0.55	0.57	0.55	0.85	0.76
BLCA	RNASeq	HiSeqV2	ML	SVM	0.94	0.95	0.94	0.94	0.97	0.99
BLCA	RNASeq	HiSeqV2-PANCAN	ML	SVM	0.94	0.95	0.94	0.94	0.97	0.99
BLCA	Exon	HiSeqV2-exon	ML	HGB	0.89	0.94	0.89	0.9	0.98	0.99
BLCA	RNASeq	HiSeqV2-percentile	ML	CNB	0.93	0.95	0.93	0.93	0.97	0.98
BLCA	RPPA	RPPA	DL	CNN	0.77	0.78	0.77	0.77	0.93	0.88
BLCA	RPPA	RPPA-RBN	ML	XGB	0.76	0.87	0.76	0.77	0.94	0.89
BLCA	SNP	mc3-gene-level	DL	RESNET152	0.39	0.36	0.39	0.36	0.82	0.55

BLCA	miRNA	miRNA-HiSeq-gene	DL	RESNET18	0.97	0.97	0.97	0.97	0.99	1
BRCA	Array	AgilentG4502A-07-3	ML	BNB	0.87	0.77	0.87	0.8	0.96	0.96
BRCA	Meth.	HumanMethylation27	ML	HGB	0.76	0.86	0.76	0.79	0.92	0.93
BRCA	Meth.	HumanMethylation450	ML	GNB	0.642	0.593	0.642	0.603	0.902	0.79
BRCA	CNV	Gistic2-all-data-by-genes	ML	GNB	0.65	0.58	0.65	0.57	0.9	0.79
BRCA	CNV	Gistic2-all-thresholded	ML	GNB	0.59	0.52	0.59	0.49	0.88	0.77
BRCA	RNASeq	HiSeqV2	DL	CNN	0.8	0.85	0.8	0.82	0.97	0.98
BRCA	RNASeq	HiSeqV2-PANCAN	DL	CNN_LSTM	0.79	0.79	0.79	0.79	0.96	0.96
BRCA	Exon	HiSeqV2-exon	ML	LR	0.81	0.84	0.81	0.82	0.96	0.99
BRCA	RNASeq	HiSeqV2-percentile	ML	LR	0.83	0.83	0.83	0.83	0.97	0.99
BRCA	RPPA	RPPA	DL	CNN_LSTM	0.65	0.63	0.65	0.64	0.93	0.9
BRCA	RPPA	RPPA-RBN	DL	RESNET34	0.7	0.74	0.7	0.71	0.93	0.9
BRCA	SNP	mc3-gene-level	ML	RF	0.37	0.25	0.37	0.29	0.88	0.68
BRCA	miRNA	miRNA-GA-gene	ML	SVM	0.88	0.81	0.88	0.83	0.95	0.94
BRCA	miRNA	miRNA-HiSeq-gene	ML	SVM	0.75	0.79	0.75	0.77	0.95	0.96
COAD	Meth.	HumanMethylation450	ML	QDA	0.93	0.88	0.93	0.89	0.93	0.93
COAD	RNASeq	GAV2	ML	LR	0.86	0.82	0.86	0.84	0.96	0.87
COAD	Exon	GAV2-exon	ML	SVM	1	1	1	1	1	1
COAD	CNV	Gistic2-all-data-by-genes	ML	RF	0.8	0.83	0.8	0.81	0.92	0.93
COAD	CNV	Gistic2-all-thresholded	ML	CB	0.8	0.82	0.8	0.8	0.92	0.93
COAD	RPPA	RPPA-RBN	ML	SVM	0.65	0.66	0.65	0.65	0.83	0.79
COAD	SNP	mc3-gene-level	DL	RESNET50	0.73	0.71	0.73	0.72	0.86	0.79
COAD	miRNA	miRNA-GA-gene	DL	DEEPGENE	0.752	0.786	0.752	0.763	0.885	0.94
ESCA	CNV	Gistic2-all-data-by-genes	DL	CNN	1	1	1	1	1	1
ESCA	CNV	Gistic2-all-thresholded	DL	DEEPGENE	1	1	1	1	1	1
ESCA	RPPA	RPPA	DL	CNN_LSTM	1	1	1	1	1	1

ESCA	SNP	mc3-gene-level	DL	MLP	0.85	0.85	0.85	0.85	0.85	0.85
GBM	Array	AgilentG4502A-07-1	DL	NN	0.73	0.77	0.73	0.74	0.93	0.94
GBM	Array	AgilentG4502A-07-2	ML	XGB	0.75	0.8	0.75	0.76	0.94	0.91
GBM	Meth.	HumanMethylation450	ML	DT	0.57	0.59	0.57	0.57	0.89	0.73
GBM	CNV	Gistic2-all-data-by-genes	ML	BNB	0.5	0.34	0.5	0.35	0.86	0.73
GBM	CNV	Gistic2-all-thresholded	ML	SVM	0.4	0.42	0.4	0.4	0.85	0.7
GBM	Array	HT-HG-U133A	ML	HGB	0.449	0.478	0.449	0.457	0.922	0.9
GBM	RPPA	RPPA-RBN	DL	MLP	0.49	0.65	0.49	0.53	0.82	0.73
GBM	SNP	mc3-gene-level	DL	RESNET18	0.37	0.41	0.37	0.34	0.81	0.71
HNSC	Meth.	HumanMethylation450	ML	CNB	0.63	0.67	0.63	0.62	0.87	0.81
HNSC	CNV	Gistic2-all-data-by-genes	ML	RF	0.64	0.65	0.64	0.64	0.89	0.82
HNSC	CNV	Gistic2-all-thresholded	ML	RF	0.51	0.52	0.51	0.51	0.84	0.69
HNSC	RPPA	RPPA	ML	GNB	0.71	0.71	0.71	0.7	0.9	0.85
HNSC	RPPA	RPPA-RBN	ML	HGB	0.53	0.55	0.53	0.53	0.84	0.76
HNSC	SNP	mc3-gene-level	ML	RF	0.38	0.3	0.38	0.31	0.79	0.64
KIRC	Meth.	HumanMethylation450	DL	RESNET101	0.34	0.25	0.34	0.27	0.83	0.66
KIRC	CNV	Gistic2-all-data-by-genes	DL	MLP	0.34	0.34	0.34	0.34	0.84	0.6
KIRC	CNV	Gistic2-all-thresholded	ML	HGB	0.34	0.32	0.34	0.33	0.85	0.57
KIRC	RPPA	RPPA	DL	DEEPGENE	0.467	0.465	0.467	0.459	0.876	0.7
KIRC	RPPA	RPPA-RBN	DL	CNN_LSTM	0.41	0.38	0.41	0.38	0.87	0.68
KIRC	SNP	mc3-gene-level	DL	RESNET50	0.27	0.16	0.27	0.2	0.83	0.55
KIRC	miRNA	miRNA-GA-gene	DL	CNN	0.539	0.55	0.539	0.536	0.909	0.81
KIRP	CNV	Gistic2-all-data-by-genes	DL	DEEPGENE	0.887	0.871	0.887	0.875	0.946	0.89
KIRP	CNV	Gistic2-all-thresholded	DL	CNN_RNN	0.74	0.72	0.74	0.73	0.9	0.94
KIRP	RPPA	RPPA	DL	MLP	0.77	0.89	0.77	0.8	0.91	0.93
KIRP	SNP	mc3-gene-level	ML	DT	0.61	0.67	0.61	0.62	0.81	0.71

LAML	miRNA	GA	ML	SVM	0.77	0.829	0.77	0.759	0.957	0.96
LAML	Exon	GA-exon	ML	LR	0.73	0.78	0.73	0.73	0.96	0.96
LAML	CNV	Gistic2-all-data-by-genes	DL	RESNET50	0.32	0.28	0.32	0.21	0.89	0.67
LAML	CNV	Gistic2-all-thresholded	ML	GNB	0.25	0.2	0.25	0.21	0.87	0.56
LAML	RNASeq	HiSeqV2	ML	RF	0.69	0.69	0.69	0.67	0.96	0.94
LAML	RNASeq	HiSeqV2-PANCAN	ML	RF	0.69	0.69	0.69	0.67	0.96	0.94
LAML	Exon	HiSeqV2-exon	ML	HGB	0.71	0.7	0.71	0.69	0.96	0.96
LAML	RNASeq	HiSeqV2-percentile	ML	HGB	0.7	0.66	0.7	0.68	0.96	0.94
LAML	miRNA	miRNA-GA-gene	ML	HGB	0.63	0.64	0.63	0.62	0.94	0.9
LAML	SNP	mutation-wustl-gene	DL	CNN	0.267	0.41	0.267	0.273	0.879	0.558
LGG	CNV	Gistic2-all-data-by-genes	DL	NN	0.65	0.65	0.65	0.65	0.97	0.89
LGG	CNV	Gistic2-all-thresholded	DL	MLP	0.66	0.58	0.66	0.61	0.97	0.91
LGG	SNP	mc3-gene-level	DL	DEEPGENE	0.633	0.669	0.633	0.645	0.964	0.95
LIHC	Meth.	HumanMethylation450	DL	LSTM	0.69	0.64	0.69	0.66	0.95	0.82
LIHC	CNV	Gistic2-all-data-by-genes	ML	LR	0.67	0.67	0.67	0.67	0.84	0.77
LIHC	CNV	Gistic2-all-thresholded	DL	LSTM	0.65	0.64	0.65	0.64	0.82	0.77
LIHC	RNASeq	HiSeqV2	ML	CB	0.69	0.66	0.69	0.67	0.95	0.81
LIHC	RNASeq	HiSeqV2-PANCAN	DL	NN	0.73	0.74	0.73	0.73	0.93	0.89
LIHC	Exon	HiSeqV2-exon	ML	CB	0.63	0.6	0.63	0.61	0.93	0.84
LIHC	RNASeq	HiSeqV2-percentile	ML	CB	0.69	0.65	0.69	0.67	0.95	0.85
LIHC	RPPA	RPPA	DL	RNN	0.45	0.45	0.45	0.42	0.84	0.78
LIHC	SNP	mc3-gene-level	ML	DT	0.46	0.464	0.46	0.382	0.809	0.635
LIHC	miRNA	miRNA-HiSeq-gene	ML	SVM	0.86	0.86	0.86	0.86	0.93	0.92
LUAD	Meth.	HumanMethylation27	DL	RESNET101	1	1	1	1	1	1
LUAD	Meth.	HumanMethylation450	ML	RF	0.79	0.83	0.79	0.8	0.96	0.94
LUAD	CNV	Gistic2-all-data-by-genes	DL	NN	0.69	0.7	0.69	0.66	0.94	0.91

LUAD	CNV	Gistic2-all-thresholded	ML	GNB	0.62	0.68	0.62	0.61	0.92	0.78
LUAD	RPPA	RPPA	ML	SVM	0.63	0.74	0.63	0.64	0.93	0.9
LUAD	RPPA	RPPA-RBN	DL	RESNET18	0.52	0.58	0.52	0.54	0.9	0.76
LUAD	SNP	mc3-gene-level	ML	LR	0.35	0.45	0.35	0.34	0.88	0.72
LUSC	Array	AgilentG4502A-07-3	ML	HGB	0.85	0.9	0.85	0.84	0.96	0.99
LUSC	CNV	Gistic2-all-data-by-genes	ML	GNB	0.59	0.59	0.59	0.58	0.87	0.75
LUSC	CNV	Gistic2-all-thresholded	ML	XGB	0.6	0.63	0.6	0.61	0.86	0.77
LUSC	Array	HT-HG-U133A	DL	RESNET34	0.92	0.97	0.92	0.93	0.98	0.92
LUSC	RPPA	RPPA	DL	RNN	0.594	0.51	0.594	0.544	0.895	0.865
LUSC	RPPA	RPPA-RBN	ML	AB	0.36	0.43	0.36	0.35	0.78	0.6
LUSC	SNP	mc3-gene-level	ML	HGB	0.4	0.4	0.4	0.39	0.79	0.59
LUSC	miRNA	miRNA-GA-gene	ML	SVM	0.67	0.57	0.67	0.61	0.92	0.9
PCPG	CNV	Gistic2-all-data-by-genes	ML	RF	0.56	0.49	0.56	0.52	0.9	0.84
PCPG	CNV	Gistic2-all-thresholded	ML	XGB	0.64	0.68	0.64	0.64	0.88	0.89
PCPG	RNASeq	HiSeqV2	DL	RESNET18	0.94	0.96	0.94	0.94	0.99	1
PCPG	RNASeq	HiSeqV2-PANCAN	DL	DEEPGENE	0.95	0.981	0.95	0.962	0.989	1
PCPG	Exon	HiSeqV2-exon	ML	SVM	0.94	0.96	0.94	0.94	0.99	0.98
PCPG	RNASeq	HiSeqV2-percentile	DL	MLP	1	1	1	1	1	1
PCPG	RPPA	RPPA	ML	SVM	0.95	0.94	0.95	0.94	0.96	0.93
PCPG	SNP	mc3-gene-level	DL	CNN_GRU	0.5	0.52	0.5	0.5	0.87	0.7
PCPG	miRNA	miRNA-HiSeq-gene	ML	HGB	0.98	0.96	0.98	0.97	0.99	1
PRAD	CNV	Gistic2-all-data-by-genes	DL	MLP	0.5	0.45	0.5	0.47	0.88	0.71
PRAD	CNV	Gistic2-all-thresholded	DL	CNN_LSTM	0.45	0.38	0.45	0.39	0.87	0.68
PRAD	RNASeq	HiSeqV2	ML	HGB	0.99	0.99	0.99	0.99	1	1
PRAD	RNASeq	HiSeqV2-PANCAN	ML	HGB	0.99	0.99	0.99	0.99	1	1
PRAD	Exon	HiSeqV2-exon	ML	XGB	0.93	0.91	0.93	0.92	0.98	0.98

PRAD	RNASeq	HiSeqV2-percentile	ML	HGB	0.93	0.98	0.93	0.95	0.99	0.99
PRAD	RPPA	RPPA	ML	SVM	0.44	0.37	0.44	0.39	0.9	0.7
PRAD	SNP	mc3-gene-level	ML	SVM	0.42	0.4	0.42	0.4	0.85	0.63
PRAD	miRNA	miRNA-HiSeq-gene	DL	MLP	0.48	0.51	0.48	0.46	0.89	0.77
SKCM	Meth.	HumanMethylation450	DL	RESNET34	0.39	0.43	0.39	0.37	0.86	0.62
SKCM	CNV	Gistic2-all-thresholded	DL	RNN	0.46	0.41	0.46	0.43	0.85	0.73
STAD	Meth.	HumanMethylation450	DL	DEEPGENE	0.838	0.819	0.838	0.827	0.933	0.93
STAD	CNV	Gistic2-all-data-by-genes	DL	CNN_GRU	0.76	0.77	0.76	0.76	0.94	0.94
STAD	CNV	Gistic2-all-thresholded	DL	CNN_GRU	0.68	0.73	0.68	0.7	0.91	0.89
STAD	RPPA	RPPA	DL	RNN	0.717	0.763	0.717	0.734	0.905	0.879
STAD	SNP	mc3-gene-level	ML	HGB	0.77	0.76	0.77	0.76	0.93	0.92
THCA	Meth.	HumanMethylation450	ML	SVM	0.67	0.65	0.67	0.65	0.96	0.81
THCA	CNV	Gistic2-all-data-by-genes	DL	MLP	0.3	0.32	0.3	0.3	0.87	0.6
THCA	CNV	Gistic2-all-thresholded	DL	LSTM	0.27	0.23	0.27	0.22	0.87	0.67
THCA	RNASeq	HiSeqV2	ML	LR	0.77	0.75	0.77	0.76	0.98	0.94
THCA	RNASeq	HiSeqV2-PANCAN	DL	CNN	0.77	0.74	0.77	0.75	0.98	0.95
THCA	Exon	HiSeqV2-exon	ML	HGB	0.83	0.91	0.83	0.85	0.98	0.99
THCA	RNASeq	HiSeqV2-percentile	DL	RESNET18	0.76	0.76	0.76	0.76	0.98	0.92
THCA	RPPA	RPPA	DL	MLP	0.54	0.53	0.54	0.53	0.93	0.82
THCA	SNP	mc3-gene-level	ML	HGB	0.32	0.16	0.32	0.21	0.88	0.67
THCA	miRNA	miRNA-HiSeq-gene	DL	RESNET18	0.687	0.689	0.687	0.68	0.965	0.934
UCEC	Meth.	HumanMethylation27	DL	LSTM	0.881	0.937	0.881	0.9	0.965	0.956
UCEC	Meth.	HumanMethylation450	ML	HGB	0.636	0.613	0.636	0.621	0.943	0.84
UCEC	CNV	Gistic2-all-data-by-genes	DL	RNN	0.48	0.49	0.48	0.47	0.89	0.77
UCEC	CNV	Gistic2-all-thresholded	DL	CNN_LSTM	0.45	0.49	0.45	0.41	0.89	0.78
UCEC	RPPA	RPPA	DL	CNN_GRU	0.573	0.555	0.573	0.56	0.918	0.813

UCEC	RPPA	RPPA-RBN	DL	GRU	0.59	0.58	0.59	0.58	0.92	0.8
UCEC	SNP	mc3-gene-level	ML	XGB	0.7	0.71	0.7	0.71	0.96	0.89
UCEC	miRNA	miRNA-GA-gene	ML	HGB	0.67	0.83	0.67	0.71	0.9	0.81

Supplementary Table S3: MACC of top performing classifier-modality combination across 20 different cancers in terms of 8 distinct omics modalities. (b) PR-RC difference of top performing classifier-modality combination for a cancer. A larger PR-RC difference indicates a higher degree of bias in the molecular subtype classification results for the corresponding cancer.

**Cancer**	**Modality**	**Scale**	**Type**	**Classifier**	**MACC**	**PR**	**RE**	**F1**	**MCC**	**AUROC**	**PR_R**
ACC	SNP	mc3-gene-level	ML	CNB	0.53	0.3	0.53	0.37	0.74	0.67	
BLCA	CNV	Gistic2-all-thresholded	DL	NN	0.48	0.54	0.48	0.49	0.82	0.72	
BRCA	Meth.	HumanMethylation450	ML	HGB	0.626	0.85	0.626	0.66	0.933	0.88	
COAD	Meth.	HumanMethylation450	ML	QDA	0.93	0.88	0.93	0.89	0.93	0.93	
ESCA	CNV	Gistic2-all-data-by-genes	DL	CNN	1	1	1	1	1	1	
GBM	Array	AgilentG4502A-07-1	ML	XGB	0.7	0.87	0.7	0.73	0.92	0.91	
HNSC	SNP	mc3-gene-level	ML	RF	0.38	0.3	0.38	0.31	0.79	0.64	
KIRC	SNP	mc3-gene-level	DL	RESNET50	0.27	0.16	0.27	0.2	0.83	0.55	
KIRP	RPPA	RPPA	DL	MLP	0.77	0.89	0.77	0.8	0.91	0.93	
LAML	SNP	mutation-wustl-gene	DL	CNN	0.267	0.41	0.267	0.273	0.879	0.558	
LGG	SNP	mc3-gene-level	DL	DEEPGENE	0.633	0.669	0.633	0.645	0.964	0.95	
LIHC	Meth.	HumanMethylation450	DL	LSTM	0.69	0.64	0.69	0.66	0.95	0.82	
LUAD	RPPA	RPPA	ML	SVM	0.63	0.74	0.63	0.64	0.93	0.9	
LUSC	miRNA	miRNA-GA-gene	ML	SVM	0.67	0.57	0.67	0.61	0.92	0.9	
PCPG	RNASeq	HiSeqV2	DL	MLP	0.61	0.88	0.61	0.63	0.87	0.99	
PRAD	RPPA	RPPA	ML	SVM	0.44	0.37	0.44	0.39	0.9	0.7	
SKCM	CNV	Gistic2-all-thresholded	DL	RNN	0.46	0.41	0.46	0.43	0.85	0.73	
STAD	CNV	Gistic2-all-thresholded	DL	CNN_GRU	0.68	0.73	0.68	0.7	0.91	0.89	
THCA	SNP	mc3-gene-level	ML	HGB	0.32	0.16	0.32	0.21	0.88	0.67	
UCEC	miRNA	miRNA-GA-gene	ML	HGB	0.67	0.83	0.67	0.71	0.9	0.81	

Supplementary Table S4: Modality-wise average MACC values for (a) ML

Modality	Classifier	MACC	PR	RE	F1	MCC	AUROC
Array	AB	0.457667	0.451667	0.457667	0.433667	0.8765	0.861667
CNV	AB	0.403846	0.396667	0.403846	0.361282	0.839487	0.738462
Exon	AB	0.665556	0.736667	0.665556	0.674444	0.926667	0.92
Meth.	AB	0.484188	0.458875	0.484188	0.447688	0.813063	0.759
RNASeq	AB	0.6224	0.65012	0.6224	0.6154	0.90512	0.86712
RPPA	AB	0.406522	0.387826	0.406522	0.375217	0.825217	0.71913
SNP	AB	0.379105	0.365263	0.379105	0.334842	0.808211	0.663211
miRNA	AB	0.472571	0.493143	0.472571	0.456214	0.857	0.811429
Array	BNB	0.536167	0.515167	0.536167	0.494833	0.877167	0.751667
CNV	BNB	0.434872	0.419487	0.434872	0.405385	0.839231	0.665641
Exon	BNB	0.468889	0.468889	0.468889	0.444444	0.872222	0.703333
Meth.	BNB	0.263125	0.124687	0.263125	0.163438	0.73625	0.518125
RNASeq	BNB	0.5328	0.5036	0.5328	0.5024	0.8884	0.842
RPPA	BNB	0.473913	0.468261	0.473913	0.453478	0.845652	0.764783
SNP	BNB	0.286368	0.177947	0.286368	0.198053	0.771053	0.561684
miRNA	BNB	0.488714	0.528143	0.488714	0.475071	0.8615	0.816429
Array	CB	0.489	0.454667	0.489	0.453833	0.9	0.901667
CNV	CB	0.435897	0.428974	0.435897	0.401795	0.851795	0.768974
Exon	CB	0.547778	0.555556	0.547778	0.52	0.905556	0.936667
Meth.	CB	0.5395	0.556625	0.5395	0.5165	0.853625	0.826
RNASeq	CB	0.606	0.6192	0.606	0.584	0.922	0.9332
RPPA	CB	0.433913	0.446522	0.433913	0.396087	0.837826	0.786522
SNP	CB	0.324737	0.235316	0.324737	0.244842	0.788421	0.655474
miRNA	CB	0.448214	0.451714	0.448214	0.409643	0.845786	0.875714
Array	CNB	0.592	0.6155	0.592	0.598	0.9335	0.835
Exon	CNB	0.597778	0.661111	0.597778	0.592222	0.915556	0.817778
Meth.	CNB	0.496813	0.4725	0.496813	0.458563	0.818188	0.759375
RNASeq	CNB	0.647529	0.676588	0.647529	0.633471	0.928353	0.881765
SNP	CNB	0.309684	0.290895	0.309684	0.250263	0.780632	0.624316
miRNA	CNB	0.551786	0.584929	0.551786	0.525929	0.877571	0.835
Array	DT	0.517333	0.536833	0.517333	0.513	0.891	0.701667
CNV	DT	0.418462	0.425128	0.418462	0.412308	0.838462	0.631538
Exon	DT	0.638889	0.641111	0.638889	0.634444	0.915556	0.774444
Meth.	DT	0.52725	0.546813	0.52725	0.523688	0.842688	0.685
RNASeq	DT	0.6572	0.6556	0.6572	0.644	0.9036	0.7812
RPPA	DT	0.400435	0.413478	0.400435	0.4	0.825217	0.613043
SNP	DT	0.412632	0.409684	0.412632	0.381684	0.821526	0.615526
miRNA	DT	0.476929	0.484857	0.476929	0.473786	0.841786	0.659286
Array	GB	0.2545	0.170167	0.2545	0.1765	0.797167	0.825
CNV	GB	0.299231	0.226667	0.299231	0.230513	0.783846	0.699487
Exon	GB	0.395556	0.365556	0.395556	0.352222	0.846667	0.904444
Meth.	GB	0.358063	0.299937	0.358063	0.30125	0.783688	0.759375

RNASeq	GB	0.3664	0.324	0.3664	0.3208	0.8212	0.8584
RPPA	GB	0.294348	0.211739	0.294348	0.221739	0.773913	0.676957
SNP	GB	0.278158	0.215053	0.278158	0.200211	0.767895	0.658842
miRNA	GB	0.313786	0.250643	0.313786	0.254643	0.795786	0.727143
Array	GNB	0.638333	0.688667	0.638333	0.639333	0.917833	0.801667
CNV	GNB	0.462821	0.476667	0.462821	0.441026	0.851282	0.669231
Exon	GNB	0.4	0.443333	0.4	0.37	0.852222	0.626667
Meth.	GNB	0.553375	0.549187	0.553375	0.534938	0.846	0.71125
RNASeq	GNB	0.3628	0.364	0.3628	0.3232	0.8308	0.598
RPPA	GNB	0.477826	0.478261	0.477826	0.458261	0.849565	0.742609
SNP	GNB	0.271263	0.223421	0.271263	0.205211	0.769	0.522737
miRNA	GNB	0.297857	0.300286	0.297857	0.277143	0.785857	0.54
Array	HGB	0.6765	0.744667	0.6765	0.687833	0.937	0.943333
CNV	HGB	0.476667	0.488974	0.476667	0.467949	0.858974	0.769487
Exon	HGB	0.777778	0.823333	0.777778	0.78	0.958889	0.963333
Meth.	HGB	0.624938	0.6215	0.624938	0.606063	0.866813	0.821875
RNASeq	HGB	0.7508	0.778	0.7508	0.7524	0.9428	0.9496
RPPA	HGB	0.49087	0.516087	0.49087	0.487391	0.861304	0.785217
SNP	HGB	0.382842	0.336789	0.382842	0.343263	0.802158	0.635368
miRNA	HGB	0.637286	0.658214	0.637286	0.634571	0.897929	0.862857
Array	KNN	0.497833	0.5645	0.497833	0.492333	0.884667	0.853333
CNV	KNN	0.404359	0.423333	0.404359	0.365385	0.829744	0.700769
Exon	KNN	0.564444	0.621111	0.564444	0.557778	0.896667	0.888889
Meth.	KNN	0.506688	0.570313	0.506688	0.48425	0.837875	0.758125
RNASeq	KNN	0.6364	0.7124	0.6364	0.6396	0.9156	0.9128
RPPA	KNN	0.468261	0.527826	0.468261	0.461739	0.848696	0.748261
SNP	KNN	0.254211	0.140579	0.254211	0.124842	0.754737	0.524526
miRNA	KNN	0.533857	0.602143	0.533857	0.525143	0.867286	0.836429
Array	LGBM	0.242833	0.145167	0.242833	0.163167	0.792167	0.86
CNV	LGBM	0.287949	0.219487	0.287949	0.214359	0.774103	0.715641
Exon	LGBM	0.382222	0.336667	0.382222	0.326667	0.847778	0.93
Meth.	LGBM	0.34125	0.265625	0.34125	0.266938	0.773625	0.780625
RNASeq	LGBM	0.3604	0.3068	0.3604	0.3092	0.826	0.8896
RPPA	LGBM	0.288261	0.204348	0.288261	0.21	0.77087	0.702609
SNP	LGBM	0.264368	0.15	0.264368	0.179632	0.761579	0.637105
miRNA	LGBM	0.301857	0.224286	0.301857	0.229786	0.791929	0.776429
Array	LR	0.672667	0.699833	0.672667	0.670667	0.930833	0.938333
CNV	LR	0.486667	0.488718	0.486667	0.475385	0.858974	0.747949
Exon	LR	0.747778	0.767778	0.747778	0.748889	0.95	0.965556
Meth.	LR	0.652313	0.702125	0.652313	0.659875	0.881063	0.83875
RNASeq	LR	0.7728	0.7928	0.7728	0.772	0.952	0.9524
RPPA	LR	0.522174	0.53913	0.522174	0.522609	0.871304	0.797826
SNP	LR	0.391789	0.370368	0.391789	0.346947	0.816895	0.691368

miRNA	LR	0.688571	0.712857	0.688571	0.685429	0.9155	0.895714
Array	QDA	0.261	0.2585	0.261	0.251	0.806167	0.533333
CNV	QDA	0.286154	0.281282	0.286154	0.238205	0.769231	0.527179
Exon	QDA	0.243333	0.235556	0.243333	0.232222	0.793333	0.517778
Meth.	QDA	0.28525	0.275375	0.28525	0.274875	0.7525	0.51875
RNASeq	QDA	0.2568	0.2532	0.2568	0.2472	0.7756	0.5156
RPPA	QDA	0.268696	0.253478	0.268696	0.246087	0.762609	0.517826
SNP	QDA	0.258421	0.203158	0.258421	0.186789	0.761105	0.507895
miRNA	QDA	0.228786	0.239929	0.228786	0.223786	0.767429	0.499286
Array	RF	0.595	0.654333	0.595	0.603	0.923667	0.908333
CNV	RF	0.487949	0.516667	0.487949	0.480769	0.86359	0.770513
Exon	RF	0.673333	0.725556	0.673333	0.676667	0.934444	0.944444
Meth.	RF	0.602063	0.632	0.602063	0.59325	0.87075	0.81375
RNASeq	RF	0.6772	0.7312	0.6772	0.6776	0.9332	0.9412
RPPA	RF	0.483478	0.508696	0.483478	0.471739	0.854783	0.775652
SNP	RF	0.392105	0.366105	0.392105	0.338842	0.812632	0.670316
miRNA	RF	0.560643	0.610857	0.560643	0.551643	0.878286	0.873571
Array	SVM	0.689	0.736167	0.689	0.690833	0.936667	0.941667
CNV	SVM	0.500513	0.495897	0.500513	0.484103	0.862564	0.746667
Exon	SVM	0.748889	0.771111	0.748889	0.748889	0.957778	0.937778
Meth.	SVM	0.637063	0.678	0.637063	0.639563	0.876688	0.81875
RNASeq	SVM	0.7812	0.8056	0.7812	0.7836	0.956	0.9388
RPPA	SVM	0.548261	0.564783	0.548261	0.545652	0.876522	0.793478
SNP	SVM	0.387579	0.363526	0.387579	0.342	0.814789	0.671053
miRNA	SVM	0.698571	0.7085	0.698571	0.694214	0.917643	0.878571
Array	XGB	0.715667	0.7625	0.715667	0.7175	0.946333	0.941667
CNV	XGB	0.479744	0.489231	0.479744	0.47359	0.86	0.778462
Exon	XGB	0.763333	0.824444	0.763333	0.774444	0.947778	0.95
Meth.	XGB	0.595313	0.62025	0.595313	0.589313	0.857	0.818125
RNASeq	XGB	0.7016	0.706	0.7016	0.696	0.9284	0.924
RPPA	XGB	0.499565	0.517391	0.499565	0.493043	0.861739	0.775652
SNP	XGB	0.404158	0.407368	0.404158	0.391158	0.817526	0.679579
miRNA	XGB	0.602786	0.641143	0.602786	0.599143	0.8815	0.859286

Supplementary Table S5: Modality-wise average MACC values for DL classifiers.

**Modality**	**Classifier**	**ACC**	**PR**	**RE**	**F1**	**MCC**	**AUROC**
Array	CNN	0.614333	0.687	0.614333	0.613	0.912	0.926667
CNV	CNN	0.488205	0.506667	0.488205	0.473846	0.864615	0.771282
Exon	CNN	0.269556	0.156222	0.269556	0.180444	0.807667	0.603667
Meth.	CNN	0.5495	0.556438	0.5495	0.511	0.859	0.79375
RNASeq	CNN	0.64808	0.64724	0.64808	0.62692	0.9192	0.9104

RPPA	CNN	0.521217	0.554826	0.521217	0.521261	0.868391	0.777435
SNP	CNN	0.351211	0.342263	0.351211	0.312895	0.804789	0.653053
miRNA	CNN	0.605214	0.645429	0.605214	0.597286	0.895643	0.888786
Array	CNN_GRU	0.286167	0.210167	0.286167	0.2115	0.8105	0.778333
CNV	CNN_GRU	0.476154	0.486154	0.476154	0.464103	0.863846	0.762564
Exon	CNN_GRU	0.214444	0.085222	0.214444	0.119778	0.785556	0.48
Meth.	CNN_GRU	0.286875	0.13875	0.286875	0.180438	0.74875	0.619188
RNASeq	CNN_GRU	0.316	0.20048	0.316	0.22892	0.8066	0.68336
RPPA	CNN_GRU	0.492478	0.504261	0.492478	0.473435	0.857	0.765174
SNP	CNN_GRU	0.384368	0.363263	0.384368	0.353316	0.814684	0.649474
miRNA	CNN_GRU	0.415429	0.346357	0.415429	0.360286	0.8385	0.777286
Array	CNN_LSTM	0.257833	0.1435	0.257833	0.173167	0.797167	0.658333
CNV	CNN_LSTM	0.468462	0.460256	0.468462	0.439744	0.859487	0.757692
Exon	CNN_LSTM	0.214111	0.085889	0.214111	0.120111	0.785889	0.493889
Meth.	CNN_LSTM	0.29	0.163188	0.29	0.189188	0.754375	0.512
RNASeq	CNN_LSTM	0.27184	0.1358	0.27184	0.1726	0.78616	0.63364
RPPA	CNN_LSTM	0.505652	0.512043	0.505652	0.497783	0.864217	0.784174
SNP	CNN_LSTM	0.344368	0.333947	0.344368	0.292895	0.805579	0.649737
miRNA	CNN_LSTM	0.398643	0.3295	0.398643	0.330571	0.821429	0.808786
Array	CNN_RNN	0.307833	0.215167	0.307833	0.234833	0.817167	0.678333
CNV	CNN_RNN	0.495128	0.504103	0.495128	0.485128	0.866923	0.761795
Exon	CNN_RNN	0.214111	0.084444	0.214111	0.120111	0.785889	0.498444
Meth.	CNN_RNN	0.29875	0.165	0.29875	0.199813	0.76375	0.641125
RNASeq	CNN_RNN	0.33252	0.257	0.33252	0.2498	0.81024	0.75312
RPPA	CNN_RNN	0.520261	0.544304	0.520261	0.517565	0.869696	0.785087
SNP	CNN_RNN	0.368	0.33	0.368	0.325737	0.809211	0.655211
miRNA	CNN_RNN	0.450714	0.399786	0.450714	0.401	0.847857	0.823286
Array	DEEPGENE	0.460333	0.474167	0.460333	0.4445	0.879167	0.826667
CNV	DEEPGENE	0.445763	0.455974	0.445763	0.431605	0.850789	0.726053
Exon	DEEPGENE	0.25	0.09	0.25	0.132	0.75	0.529
Meth.	DEEPGENE	0.463733	0.481733	0.463733	0.4176	0.8256	0.738
RNASeq	DEEPGENE	0.52136	0.50688	0.52136	0.49348	0.87156	0.80448
RPPA	DEEPGENE	0.447045	0.470455	0.447045	0.436545	0.835409	0.715273
SNP	DEEPGENE	0.396789	0.387368	0.396789	0.371789	0.820737	0.662947
miRNA	DEEPGENE	0.614214	0.64	0.614214	0.606357	0.897286	0.880214
Array	DENSENET121	0.302333	0.266333	0.302333	0.239333	0.811	0.653333
CNV	DENSENET121	0.328205	0.280256	0.328205	0.27359	0.785897	0.62
Exon	DENSENET121	0.233778	0.183667	0.233778	0.175111	0.786	0.518222
Meth.	DENSENET121	0.32275	0.307313	0.32275	0.256813	0.780813	0.597438
RNASeq	DENSENET121	0.334	0.30024	0.334	0.27136	0.80636	0.68048
RPPA	DENSENET121	0.360739	0.352957	0.360739	0.327174	0.807304	0.638783
SNP	DENSENET121	0.283895	0.182	0.283895	0.178632	0.765	0.580263
miRNA	DENSENET121	0.344429	0.319	0.344429	0.285643	0.801143	0.6535

Array	DENSENET161	0.299667	0.276667	0.299667	0.228667	0.815667	0.7
CNV	DENSENET161	0.332564	0.271538	0.332564	0.262051	0.790513	0.628205
Exon	DENSENET161	0.231778	0.173889	0.231778	0.151444	0.788444	0.519444
Meth.	DENSENET161	0.294313	0.2295	0.294313	0.2275	0.749813	0.552313
RNASeq	DENSENET161	0.34736	0.33696	0.34736	0.27748	0.80864	0.66196
RPPA	DENSENET161	0.323783	0.306348	0.323783	0.295478	0.784174	0.580739
SNP	DENSENET161	0.2545	0.137278	0.2545	0.157278	0.766944	0.554611
miRNA	DENSENET161	0.336571	0.343429	0.336571	0.274	0.796643	0.651
Array	DENSENET169	0.335333	0.3145	0.335333	0.280333	0.830333	0.691667
CNV	DENSENET169	0.331282	0.277179	0.331282	0.264872	0.792308	0.627179
Exon	DENSENET169	0.231111	0.153	0.231111	0.157667	0.788889	0.532111
Meth.	DENSENET169	0.328563	0.324063	0.328563	0.285313	0.7745	0.563125
RNASeq	DENSENET169	0.35696	0.30948	0.35696	0.27936	0.81084	0.6792
RPPA	DENSENET169	0.343565	0.357478	0.343565	0.315652	0.790913	0.599217
SNP	DENSENET169	0.292263	0.206737	0.292263	0.199895	0.774579	0.592684
miRNA	DENSENET169	0.366571	0.343714	0.366571	0.316214	0.807143	0.690571
Array	DENSENET201	0.312833	0.3075	0.312833	0.256667	0.818333	0.691667
CNV	DENSENET201	0.332821	0.285385	0.332821	0.270769	0.791538	0.624359
Exon	DENSENET201	0.225333	0.189111	0.225333	0.143333	0.789	0.526778
Meth.	DENSENET201	0.267	0.228063	0.267	0.187563	0.738813	0.543813
RNASeq	DENSENET201	0.34416	0.29472	0.34416	0.27232	0.8062	0.67076
RPPA	DENSENET201	0.330261	0.308043	0.330261	0.302174	0.793217	0.606696
SNP	DENSENET201	0.282889	0.200278	0.282889	0.190222	0.776722	0.533778
miRNA	DENSENET201	0.361929	0.350571	0.361929	0.309571	0.809	0.682786
Array	DENSENET264	0.343	0.282333	0.343	0.286667	0.8265	0.66
CNV	DENSENET264	0.320657	0.269114	0.320657	0.253486	0.799143	0.630543
Exon	DENSENET264	0.238667	0.176667	0.238667	0.176111	0.790444	0.505778
Meth.	DENSENET264	0.29	0.251063	0.29	0.234375	0.743	0.561438
RNASeq	DENSENET264	0.36528	0.3286	0.36528	0.29932	0.81436	0.68588
RPPA	DENSENET264	0.344957	0.315609	0.344957	0.309304	0.800739	0.608478
SNP	DENSENET264	0.273833	0.169	0.273833	0.183722	0.772	0.547389
miRNA	DENSENET264	0.341643	0.294	0.341643	0.268286	0.802	0.659429
Array	GRU	0.4895	0.5285	0.4895	0.469833	0.8755	0.823333
CNV	GRU	0.481282	0.497949	0.481282	0.478974	0.863333	0.772051
Exon	GRU	0.214444	0.085222	0.214444	0.119778	0.785556	0.523444
Meth.	GRU	0.576	0.606938	0.576	0.559	0.864313	0.821938
RNASeq	GRU	0.37424	0.2974	0.37424	0.30588	0.82376	0.65496
RPPA	GRU	0.504	0.495696	0.504	0.482435	0.863348	0.800348
SNP	GRU	0.315316	0.296105	0.315316	0.280316	0.788263	0.613526
miRNA	GRU	0.550714	0.572214	0.550714	0.536357	0.874	0.862571
Array	LSTM	0.472833	0.481833	0.472833	0.444833	0.872167	0.788333
CNV	LSTM	0.491538	0.510513	0.491538	0.484103	0.866923	0.785385
Exon	LSTM	0.214444	0.085222	0.214444	0.118667	0.785556	0.551444

Meth.	LSTM	0.554375	0.52825	0.554375	0.520813	0.852	0.811
RNASeq	LSTM	0.36944	0.2886	0.36944	0.30228	0.82096	0.66792
RPPA	LSTM	0.461087	0.462261	0.461087	0.434826	0.849522	0.784435
SNP	LSTM	0.303053	0.258947	0.303053	0.248789	0.782947	0.602263
miRNA	LSTM	0.495643	0.484429	0.495643	0.466286	0.862571	0.869929
Array	MLP	0.596167	0.650167	0.596167	0.583167	0.908833	0.905
CNV	MLP	0.482564	0.49	0.482564	0.473846	0.858718	0.744872
Exon	MLP	0.412222	0.395556	0.412222	0.362222	0.861111	0.728889
Meth.	MLP	0.570438	0.568563	0.570438	0.55125	0.854625	0.815625
RNASeq	MLP	0.5564	0.5652	0.5564	0.5272	0.8944	0.8172
RPPA	MLP	0.541739	0.555217	0.541739	0.536957	0.877826	0.814348
SNP	MLP	0.365474	0.352947	0.365474	0.327579	0.810579	0.642211
miRNA	MLP	0.602643	0.626786	0.602643	0.591	0.885929	0.870714
Array	NN	0.6795	0.712833	0.6795	0.684333	0.931	0.933333
CNV	NN	0.508718	0.503333	0.508718	0.493077	0.866923	0.764615
Exon	NN	0.621222	0.640111	0.621222	0.610889	0.907556	0.859333
Meth.	NN	0.632563	0.657188	0.632563	0.630438	0.872188	0.816125
RNASeq	NN	0.73872	0.75164	0.73872	0.73876	0.94096	0.93992
RPPA	NN	0.428261	0.421304	0.428261	0.39813	0.832	0.76487
SNP	NN	0.311842	0.323105	0.311842	0.297474	0.784947	0.570474
miRNA	NN	0.555143	0.607071	0.555143	0.554071	0.876	0.820571
Array	RESNET101	0.454833	0.470833	0.454833	0.4075	0.867833	0.788333
CNV	RESNET101	0.350769	0.349231	0.350769	0.296667	0.799744	0.658718
Exon	RESNET101	0.332778	0.308889	0.332778	0.275111	0.829111	0.744556
Meth.	RESNET101	0.556	0.596188	0.556	0.538438	0.861625	0.8215
RNASeq	RESNET101	0.56368	0.61524	0.56368	0.54948	0.88688	0.88236
RPPA	RESNET101	0.364391	0.376826	0.364391	0.348478	0.806435	0.645913
SNP	RESNET101	0.290667	0.214	0.290667	0.224722	0.779833	0.595111
miRNA	RESNET101	0.542643	0.562857	0.542643	0.522643	0.876786	0.828
Array	RESNET152	0.411167	0.463	0.411167	0.387667	0.855167	0.781667
CNV	RESNET152	0.332821	0.302821	0.332821	0.273333	0.791795	0.636923
Exon	RESNET152	0.296889	0.254222	0.296889	0.235667	0.812889	0.731
Meth.	RESNET152	0.446125	0.473438	0.446125	0.406688	0.804625	0.733563
RNASeq	RESNET152	0.48024	0.47296	0.48024	0.42516	0.85676	0.82608
RPPA	RESNET152	0.318783	0.315565	0.318783	0.285348	0.782565	0.596391
SNP	RESNET152	0.308842	0.237211	0.308842	0.250632	0.779421	0.590368
miRNA	RESNET152	0.512	0.531071	0.512	0.475643	0.857286	0.791214
Array	RESNET18	0.599	0.656333	0.599	0.604667	0.918	0.91
CNV	RESNET18	0.359231	0.363846	0.359231	0.320513	0.807692	0.658205
Exon	RESNET18	0.534667	0.575	0.534667	0.497556	0.883556	0.911222
Meth.	RESNET18	0.614	0.656063	0.614	0.606875	0.873563	0.831313
RNASeq	RESNET18	0.689	0.72656	0.689	0.687	0.93416	0.93584
RPPA	RESNET18	0.481435	0.526087	0.481435	0.481304	0.847261	0.747174

SNP	RESNET18	0.322421	0.274211	0.322421	0.263211	0.788895	0.613632
miRNA	RESNET18	0.636786	0.6475	0.636786	0.626857	0.898643	0.896786
Array	RESNET34	0.5735	0.630667	0.5735	0.567833	0.905833	0.883333
CNV	RESNET34	0.360256	0.342051	0.360256	0.313846	0.804103	0.654359
Exon	RESNET34	0.425778	0.384222	0.425778	0.353444	0.854889	0.829667
Meth.	RESNET34	0.581375	0.652188	0.581375	0.57625	0.85625	0.79975
RNASeq	RESNET34	0.63992	0.70524	0.63992	0.63104	0.91596	0.91944
RPPA	RESNET34	0.440957	0.453609	0.440957	0.428522	0.833913	0.724
SNP	RESNET34	0.323684	0.313842	0.323684	0.261368	0.773895	0.622579
miRNA	RESNET34	0.626571	0.64	0.626571	0.616714	0.899857	0.886786
Array	RESNET50	0.474667	0.497333	0.474667	0.441667	0.871667	0.828333
CNV	RESNET50	0.364359	0.358205	0.364359	0.316154	0.806154	0.65
Exon	RESNET50	0.429222	0.400556	0.429222	0.37	0.843778	0.821778
Meth.	RESNET50	0.539563	0.561625	0.539563	0.515625	0.846313	0.804125
RNASeq	RESNET50	0.62832	0.70364	0.62832	0.6294	0.9122	0.92408
RPPA	RESNET50	0.408522	0.476783	0.408522	0.40713	0.81887	0.697565
SNP	RESNET50	0.341526	0.302316	0.341526	0.280368	0.790842	0.626632
miRNA	RESNET50	0.567286	0.610071	0.567286	0.563071	0.88	0.863929
Array	RNN	0.5045	0.5435	0.5045	0.4915	0.8755	0.76
CNV	RNN	0.492564	0.502821	0.492564	0.485897	0.864872	0.767949
Exon	RNN	0.214444	0.085222	0.214444	0.119778	0.785556	0.507778
Meth.	RNN	0.495625	0.473625	0.495625	0.454125	0.839	0.782063
RNASeq	RNN	0.37892	0.29988	0.37892	0.31316	0.8224	0.65336
RPPA	RNN	0.561478	0.580174	0.561478	0.555783	0.884957	0.821261
SNP	RNN	0.361421	0.370368	0.361421	0.340737	0.808632	0.655368
miRNA	RNN	0.556	0.593643	0.556	0.545071	0.8835	0.856929
